# Root zone microbial communities of *Artemisia ordosica* Krasch. at different successional stages in Mu US Sandy Land: a metagenomic perspective with culturomics insights

**DOI:** 10.3389/fmicb.2025.1585700

**Published:** 2025-05-09

**Authors:** Wen Zhu, Shuo Han, Yao Cheng, Zhihong Yu, Guozhu Zhao, Xiangwei He

**Affiliations:** ^1^College of Biological Sciences and Technology, Beijing Forestry University, Beijing, China; ^2^National Engineering Research Center of Tree Breeding and Ecological Restoration, Beijing Forestry University, Beijing, China

**Keywords:** *Artemisia ordosica* Krasch., root zone microbiota, high-throughput sequencing, successional stages, land phytoremediation

## Abstract

Phytoremediation offers a promising strategy for addressing the global challenge of land desertification. In the Mu Us Sandy Land of China, *Artemisia ordosica* Krasch. has emerged as a key species for desertification control. Its root-associated microbial communities may enhance the plant’s adaptability to sandy, nutrient-poor environments. Despite their ecological significance, comprehensive investigations of these microbial communities remain limited. In this study, microbial communities in the root zone (i.e., rhizosphere soil, non-rhizosphere soil, and root endosphere) of *A. ordosica* were analyzed via high-throughput sequencing and different isolation approaches across successional stages (moving dunes, semi-fixed dunes, and fixed dunes) in the Mu Us Sandy Land of northern China. Metagenomic analysis revealed that microbial diversity was significantly higher in the rhizosphere and non-rhizosphere soils than in the root endosphere; moving dunes exhibited lower diversity than semi-fixed and fixed dunes. Meanwhile, distinct microbial community structures across successional stages were revealed by principal coordinates analysis (PCoA), demonstrating substantial differences between the root endosphere and other zones. Environmental factors, including nitrate nitrogen (NO_3_^−^-N), organic matter (OM), available potassium (AK), and total potassium (TK), significantly influenced microbial community composition. Moreover, dominant genera such as *Arthrobacter* and *Paraphoma* were identified, potentially contributing to *A. ordosica* growth. From a culturomics perspective, 93 bacterial isolates were obtained using conventional streak plate and colony pick methods, with Firmicutes (37.63%) and *Bacillus* (23.66%) identified as the dominant taxa. In parallel, 14 fungal strains were isolated, primarily belonging to *Penicillium* (35.71%) and *Aspergillus* (21.43%), both of which are well-documented for their stress tolerance in arid ecosystems. A high-throughput cultivation and identification method, tailored to recover rare and slow-growing bacteria, was employed and successfully broadened the cultured diversity to include Proteobacteria (46.43%) and representatives of the rarely cultivated Deinococcus-Thermus phylum. This study provides metagenomic with culturomics insights into the microbial communities associated with *A. ordosica*, enhancing the understanding of plant–microbe interactions in sandy land ecosystems.

## 1 Introduction

Land desertification is among the most pressing global environmental challenges, with reports indicating increasing negative ecological and socioeconomic impacts worldwide (Huang et al., 2016). China is one of the countries most severely affected, with desertified land comprising 17.93% of its total area. However, its aggressive anti-desertification policies are designed to achieve a reversal of desertification by 2030 (Xu and Zhang, 2021). Phytoremediation is considered an environmentally sustainable approach to restoring land affected by desertification (Poria et al., 2022). *Artemisia ordosica* Krasch., a psammophytic shrub species, is widely utilized in desertification control, particularly through aerial seed dispersal (Qi, 1998). As the dominant species of the most widely distributed psammophytic plant community in the Mu Us Sandy Land (MUSL) of northern China, it effectively reduces wind erosion and contributes to sand stabilization, thereby supporting desertification restoration efforts in China (Zhang, 1994; Pang et al., 2022). *Artemisia ordosica* thrives in nutrient-poor sandy soils and may recruit specific microbial communities that promote growth and enhance stress resistance (Brisbin et al., 2022; Schmitz et al., 2022; Acuna et al., 2023). However, most research concerning plant-associated microbial recruitment has focused on cash crops, food crops, and model plants; no studies have been conducted on *A. ordosica* (Wang et al., 2020; Biget et al., 2021; Upadhyay et al., 2022). Consequently, the composition of microbial communities recruited by *A. ordosica* at different successional stages in MUSL remains unknown.

High-throughput sequencing technology has enabled the identification of uncultured microorganisms and assessment of microbial diversity in environmental samples (Bartram et al., 2011; Caporaso et al., 2012). Briefly, these methods involve the isolation and amplification of DNA from samples, the generation of large numbers of base sequences through chemical catalysis and other processes, and the subsequent analysis and processing of sequence data using computational tools to obtain biomolecular sequence information (Reuter et al., 2015). Due to its high efficiency and accuracy, high-throughput sequencing has become an essential research tool in molecular ecology (Hu et al., 2021; Liu et al., 2021). In this study, the root endosphere, rhizosphere, and non-rhizosphere microbiomes of *A. ordosica* growing in sandy soils at different successional stages in MUSL were systematically investigated via high-throughput sequencing technology. First, the structure and composition of microbial communities in the *A. ordosica* endosphere, rhizosphere, and non-rhizosphere were examined. Second, differences in these microbial communities across sandy soils at various successional stages in MUSL and the key drivers of such variations were analyzed. The findings provided a detailed understanding of the complex bacterial communities associated with sandy land plants, which may be critical for the continued application of beneficial bacteria in sandy land phytoremediation. However, these sequencing methods generate only statistical information regarding sample microbial communities and do not facilitate the cultivation of microorganisms, making in-depth research challenging. To address this limitation, culturable microorganisms from *A. ordosica* root zone samples were isolated.

The conventional streak plate method is widely used to culture microorganisms by artificially supplying the nutrients required for growth (Felsenfeld et al., 1951; Wesley et al., 1983). This method is easy to perform and remains indispensable in microbiological research. However, it has several limitations, including an inability to fully replicate actual environmental conditions during cultivation and the challenge of preventing microbial competition, which impedes the growth of slow-growing or oligotrophic species (Lewis et al., 2021). Accordingly, only a small percentage of microbes can be cultivated (Hofer, 2018). Additionally, the streak plate method is time-consuming, requires substantial material resources, and has low efficiency. In 2021, a high-throughput cultivation and identification method was introduced, combining high-throughput sequencing with microbial culture (Zhang et al., 2021). This method involves the even distribution of limiting dilution cultures across the wells of 96-well cell culture plates, maximizing the probability of obtaining single-well pure cultures High-throughput sequencing is then used to identify the resulting cultures, preventing microbial competition during cultivation and yielding a more diverse collection of microbial isolates and particularly facilitating the isolation of rare and slow-growing bacterial strains. This approach previously facilitated the isolation of 276 bacterial strains, including 62 strains of bacilli (Xia et al., 2024). To maximize culture recovery and retain diverse microbial resources, two methods were utilized in this study to isolate culturable microorganisms from the *A. ordosica* root zone: the high-throughput cultivation and identification method and the streak plate method.

High-throughput sequencing combined with microbial culture was used to analyze microbial community structure in the root zone of *A. ordosica* at different successional stages in MUSL. Both macro-level community composition and the taxonomic distribution of culturable microbial resources were examined. Culture resources were separated and preserved, establishing a foundation for future research on microbial functions and molecular mechanisms of action. The advantages of the high-throughput cultivation and identification method were also assessed, providing support for the broader application of this advanced culture technology.

## 2 Methods

### 2.1 Collection and storage of root zone samples

Samples were collected from a site in the Mu Us Sandy Land (MUSL) in Yanchi, Ningxia, China (37°44′N; 107°24′E), which covers approximately 39,385 km^2^ and constitutes 3.6% of China’s total sand region. The site has a continental monsoon climate, with an average annual temperature of 6°C–10°C, annual precipitation of 530 mm, and an average altitude of 2,754 m. Sampling was conducted at the end of October 2022 across three different vegetation successional stages in MUSL (Supplementary Figure 1): moving dunes (C), semi-fixed dunes (SS), and fixed dunes (FS). In each sampling area, three quadrats measuring 10 m × 10 m were established. *Artemisia ordosica* was uniformly distributed within each quadrat, with a minimum spacing of 10 m between individual plants. A five-point sampling strategy was utilized to collect root endosphere (O) samples (diameter < 2 mm), rhizosphere soil (R), and non-rhizosphere soil (F) from *A. ordosica* plants at a depth of 30 cm. Collection was performed via previously described methods (Beckers et al., 2017; Xiao et al., 2017). In total, 54 samples (3 succession types × 3 root location types × 6 biological replicates) were obtained (Table 1). Soil samples were passed through a 2-mm sieve and divided into two subsamples: one stored at −20°C for microbial analysis and the other used for physical and chemical property assessments.

**Table 1:** Sample information.

Sample	Successional stages	Root zone type
SS_F	Semi-fixed dunes	Non-rhizosphere soil
SS_R	Semi-fixed dunes	Rhizosphere soil
SS_O	Semi-fixed dunes	Root endosphere
FS_F	Fixed dunes	Non-rhizosphere soil
FS_R	Fixed dunes	Rhizosphere soil
FS_O	Fixed dunes	Root endosphere
C_F	Moving dunes	Non-rhizosphere soil
C_R	Moving dunes	Rhizosphere soil
C_O	Moving dunes	Root endosphere

“C” represents moving dunes, “SS” represents semi-fixed dunes, and “FS” represents fixed dunes. SS_F represents non-rhizosphere soil sample collected from semi-fixed dunes, SS_R represents rhizosphere soil sample collected from semi-fixed dunes, SS_O represents root endosphere sample collected from semi-fixed dunes, FS_F represents non-rhizosphere soil sample collected from fixed dunes, and FS_R, FS_O, C_F, C_R, and C_O are similarly defined. Sampling sites were located in the Mu Us Sandy Land near Yanchi Salt Lake, Ningxia, China, as well as the Observation and Research Station of Soil and Water Conservation at Beijing Forestry University.

### 2.2 Determination of soil physical and chemical properties

Soil pH was measured using a pH meter. Total nitrogen (TN), total phosphorus (TP), ammonium nitrogen (NH_4_^+^-N), and available phosphorus (AP) were quantified using a fully automated chemical analyzer (Smartchem 200; AMS Alliance, Rome, Italy). Nitrate nitrogen (NO_3_^−^-N) content was determined by UV–Vis spectrophotometry (Navone, 1964). Soil organic matter (OM) content was measured using the volumetric method (Walkley and Black, 1934). Total potassium (TK) was quantified by atomic absorption spectrophotometry, and available potassium (AK) was determined by flame photometry (Isaac and Kerber, 1971).

### 2.3 DNA extraction and sequencing

Genomic DNA was extracted from a total of nine root samples, nine rhizosphere soil samples, and nine non-rhizosphere soil samples using the cetyltrimethylammonium bromide method and stored at −80°C. The bacterial 16S rRNA V3–V4 region was amplified by polymerase chain reaction (PCR) using the forward primer 341F (5′-ACTCCTACGGGAGGCAGCAG-3′) and the reverse primer 806R (5′-GACTACHVGGGTATCTAATCC-3′) (Yergeau et al., 2015). The fungal internal transcribed spacer 1 (ITS1) region was amplified by PCR using the forward primer ITS5-1737F (5′-GGAAGTAAAAGTCGTAACAAGG-3′) and the reverse primer ITS2-2043R (5′-GCTGCGTTCTTCATCGATGC-3′) (Liu et al., 2017). PCR amplification and sequencing were performed by Shanghai Majorbio Bio-pharm Technology Co., Ltd. using the Illumina MiSeq PE300 platform for high-throughput sequencing (Illumina, San Diego, CA, United States).

### 2.4 Molecular biological analysis and statistical analysis

Paired-end reads from Illumina sequencing were first processed through quality control steps: raw sequences were trimmed using a sliding window approach (10 bp window size, truncating reads from window start positions if average Q-score < 20) and filtered to discard reads shorter than 50 bp. Subsequently, reads were assembled via overlapping regions using FLASH software (Magoc and Salzberg, 2011) with a minimum overlap of 10 bp and maximum mismatch tolerance rate of 0.2. The QIIME system (Caporaso et al., 2010) was then employed to demultiplex sequences based on exact barcode matches (zero mismatches) and primers (≤2 mismatches), followed by chimera removal using UCHIME (Haas et al., 2011). Clean reads were clustered into operational taxonomic units (OTUs) at 97% sequence similarity using UPARSE, based on the SILVA132 (Quast et al., 2013) and UNITE (Abarenkov et al., 2010) databases. The OTU table was randomly resampled to match the sequencing depth of the sample with the lowest read count, ensuring uniform sequencing depth across all samples. The resampled OTU table was then used for further analysis. Data were organized using Excel 2010 (Microsoft, Redmond, WA, United States). Soil physicochemical parameters were analyzed by one-way analysis of variance (ANOVA) with Tukey’s test in SPSS (version 19.0; SPSS, Chicago, IL, United States). Alpha diversity (Shannon index) was calculated using R^1^ with vegan package; the relative abundances of OTUs at the phylum and genus levels were examined using R with dplyr package. Plots were generated using GraphPad Prism 9.0.0 (GraphPad Software, San Diego, CA, USA). The Bray-Curtis distance was selected as the metric to explore variations in microbial community structure using principal coordinates analysis (PCoA). The online TUTU analysis platform^2^ was used to visualize raw data tables or abundance data tables using its built-in algorithms. Based on the user’s selected analysis method, it processes and visualizes the data, such as performing PCoA analysis using the Bray-Curtis test. While visualizing the data, the TUTU platform can also provide data tables related to the result graphs for some analyses. For example, during heatmap analysis, it can provide tables of relative abundance and average abundance for users to analyze. A Venn diagram was generated to illustrate genera shared between groups, regardless of their relative abundances. Relationships between environmental conditions and microbial community structure were examined using the Mantel test in R using linkET package, ggplot2 package and dplyr package. Co-occurrence network analysis was performed in R using the igraph package; data were filtered using a cutoff value of 0.65 and an absolute r value of 0.65 (*p* < 0.05). Gephi v. 0.9.7 was used to visualize networks (Bastian et al., 2009). Relationships between soil environmental parameters and microbial community composition were assessed using Spearman correlation heat maps and redundancy analysis (RDA) via the TUTU analysis platform (see Footnote 2).

### 2.5 Bacterial and fungal conventional isolation

Root samples (1 g per *A. ordosica* specimen) were soaked in 75% ethanol for 3 min and rinsed five times with sterile water. Next, each sample was sequentially treated with a 3% sodium hypochlorite solution for 7, 5, and 3 min, followed by five additional rinses with sterile water. The sterilized roots were then ground in 1.5 mL of sterile water using a mortar. Samples were shaken at 175 rpm at 30°C for approximately 30 min, then incubated at room temperature for 30 s. The resulting plant tissue stock solution was serially diluted to 10^−1^, 10^−2^, 10^−3^, 10^−4^, and 10^−5^ of the original concentration, producing five dilutions. Blank control 1 was prepared by spreading sterile water from the final rinse, which refers to the last step of the sterilization process, onto the plate to confirm that any observed growth did not result from residual substances. Blank control 2 was prepared by rolling an *A. ordosica* root over the plate surface several times to ensure that untreated roots did not introduce microbial contaminants. The absence of growth on both blank control plates confirmed that all bacteria cultured from root tissue fluid had originated from the root endosphere. Rhizosphere and non-rhizosphere soil samples were prepared by mixing 10 g of soil with 90 mL of sterile water, adding a few glass beads, and shaking the mixture for 30 min. The resulting soil suspension was serially diluted to 10^−2^, 10^−3^, 10^−4^, and 10^−5^, producing five dilutions.

For the isolation of bacterial strains, 200 μL of each dilution were evenly spread onto 90-mm tryptic soy broth (TSB) agar plates, with three replicates per treatment. The plates were incubated at 37°C for 48 h, after which colony growth was examined. Single colonies were selected from plates with uniform colony distribution and inoculated onto fresh medium based on morphological characteristics such as shape, size, and color. The three-line streak method was used to obtain pure bacterial colonies.

The fungal strains were isolated from the same environmental samples. The sample pretreatment procedure was carried out according to the protocol described in bacterial isolation. To put it simply, a 200 μL continuously diluted sample is evenly coated on a 90 mm potato glucose agar (PDA) plate, in three times. All petri dishes were incubated at 27°C for 5 days to monitor mycelium development. Then, different fungal colonies were selected according to the macro characteristics (colony morphology, pigmentation and edge characteristics), and one kind was picked by sterile inoculation needle and placed on fresh PDA medium for aseptic culture.

### 2.6 16S rRNA gene extraction and sequencing from purified strains

Bacterial DNA was extracted from purified bacterial strains using a TIANamp Bacteria DNA Kit (TIANGEN BIOTECH, CO., LTD, BEIJING) in accordance with the manufacturer’s instructions. For most bacterial strains, DNA extraction was performed using the boiling method: Scrape the mycelium from 1 to 2 inoculation loops and add it to 150 μL of triple-distilled water, mix well. Boil the mixed mycelium solution at 100°C for 10 min, then centrifuge at 12,000 rpm for 10 min. Collect the supernatant, which is the crude DNA solution. PCR amplification was performed under the following conditions: 95°C for 5 min, followed by 30 cycles of 94°C for 30 s, 50°C for 30 s, and 72°C for 30 s, with a final extension at 72°C for 10 min using the universal bacterial primers 27F (5′-AGRGTTYGATYMTGGCTCAG-3′) and 1492R (5′-RGYTACCTTGTTACGACTT-3′) (Stackebrandt and Goodfellow, 1991). Fungal DNA was extracted from purified bacterial strains using a E.Z.N.A. SP Fungal DNA Kit (Shanghai solarbio Bioscience & Technology Co., LTD, Shanghai) in accordance with the manufacturer’s instructions. PCR amplification was performed under the following conditions: 94°C for 10 min, followed by 35 cycles of 94°C for 15 s, 55°C for 30 s, and 72°C for 40 s, with a final extension at 72°C for 7 min using the universal ITS1 (5′-TCCGTAGGTGAACCTGCGG-3′) and ITS4 (5′-TCCTCCGCTTATTGATATGC-3′).

The PCR products of bacteria and fungi were subjected to 1% agarose gel electrophoresis, with D2000 DNA Marker (Sangon, Shanghai, China) as the size marker. Aliquots of 4 μL of each sample were loaded onto the gels, and nucleic acids were stained with GelRed (Biotium, Fremont, CA, United States). Electrophoresis was performed at 120 V for 20 min (Sub-Cell GT System, Bio-Rad, Hercules, CA, United States). Electrophoresis results were visualized using a gel imaging system (Tanon Science & Technology Co., Ltd., Shanghai, China). PCR products with positive electrophoresis results were sequenced by Beijing Ruibo Biotechnology Co., Ltd. (Beijing, China) using the sequencing primers 27F and 1492R for bacteria and ITS1 for fungi. Sequence alignment was performed using the online tool EzBioCloud^3^ (Yoon et al., 2017). All 301 obtained bacterial sequences were deposited in GenBank under accession numbers PV428570-PV428870 and fungal sequences were deposited in GenBank under accession numbers PV426714-PV426727 (Supplementary Table 1).

### 2.7 High-throughput cultivation and identification of root zone bacteria

Root-associated bacteria from *A. ordosica* across successional stages were isolated by high-throughput cultivation using a modified version of a previously described protocol (Zhang et al., 2021). The key modifications were as follows:

#### 2.7.1 Sample preparation

Root tissues were surface sterilized with 75% ethanol (30 s) and 8% sodium hypochlorite (5 min), then homogenized in 10% MgCl_2_. Soil suspensions were prepared from non-rhizosphere samples.

#### 2.7.2 Gradient dilution strategy

Suspensions were serially diluted in 10% TSB medium (100 × to 25,000×) and inoculated into 96-well plates (160 μL/well). Three technical replicates per dilution were performed across 48 plates, with sterile medium serving as the control.

#### 2.7.3 Cultivation and selection

After sealing with Parafilm, plates were incubated at 25°C for 14 days. Optimal dilution concentrations (ODCs) were defined as those at which approximately 30% of wells exhibited turbidity. Subsequent dilutions (1/3 ODC, 1 ODC, and 3 ODC) were used to minimize cultivation bias.

#### 2.7.4 DNA extraction and PCR amplification

Bacterial lysates were prepared by alkaline lysis (95°C, 30 min) and neutralized. Neutralization buffer containing 40 mM Tris–HCl at pH 7.5. The 16S rRNA gene was amplified using a two-round PCR approach:

First PCR: A 400-bp fragment was amplified using primers 799F/1193R, with *Escherichia coli* DNA as a positive control.
Second PCR: Barcoding primers were used to generate a 500-bp amplicon for multiplex sequencing.

#### 2.7.5 Bioinformatics and strain preservation

Amplicon sequence variants (ASVs) were analyzed as previously described (Zhang et al., 2021). Strain saturation was confirmed using rarefaction curves. All bacterial strains in 96-well cell culture plates were preserved in 80% glycerol at -80°C for long-term storage.

## 3 Results

### 3.1 Physicochemical properties of soil

Measurements of the physical and chemical characteristics of the soil revealed significant variations across successional stages in MUSL. Notably, NO_3_^−^-N, NH_4_^+^-N, AK, TK, and OM contents significantly differed (*p* < 0.05) (Table 2). The NO_3_^−^-N content significantly decreased during succession from moving dunes to fixed dunes (*p* < 0.05), particularly between moving dunes and semi-fixed dunes, where a 91.17% reduction was observed. Conversely, NH_4_^+^-N exhibited a continuous increase along the same successional gradient. The NH_4_^+^-N content was 6.13% higher in fixed dunes than in moving dunes (*p* < 0.05). Both AK and OM significantly varied across the successional stages (*p* < 0.05), initially increasing and then declining. AK increased by 40.34% from moving dunes to semi-fixed dunes but decreased by 21.5% from semi-fixed dunes to fixed dunes. OM exhibited a more gradual change compared with AK, increasing by 5.23% before decreasing by 26.39%. In the succession from moving dunes to semi-fixed dunes, TK decreased by 9.34% (*p* < 0.05). However, no significant differences were observed in TP, AP, available nitrogen, or pH across the successional stages from moving dunes to fixed dunes. In conclusion, soil properties differed across successional stages in MUSL. Furthermore, a significant positive correlation was identified between OM and NO_3_^−^-N (*p* < 0.01) (Supplementary Figure 2). Significant negative correlations were found among NH_4_^+^-N, pH, and TP (*p* < 0.05) and between TK and AK (*p* < 0.01).

**Table 2:** Physicochemical characteristics of the soil at various successional stages in Mu Us Sandy Land.

Physicochemical properties	C	SS	FS
Nitrate nitrogen (mg/kg)	59.66 ± 1.223b	5.27 ± 0.249c	4.10 ± 0.163a
Available phosphorus (mg/kg)	2.28 ± 0.103a	2.26 ± 0.059a	2.28 ± 0.056a
Available potassium (mg/kg)	67.37 ± 0.580a	94.55 ± 1.570c	74.20 ± 0.825b
Total potassium (g/kg)	3.32 ± 0.108b	3.01 ± 0.010a	3.19 ± 0.104ab
Total phosphorus (g/kg)	0.10 ± 0.034a	0.08 ± 0.006a	0.10 ± 0.009a
Total nitrogen (%)	0.04 ± 0.005a	0.03 ± 0.007a	0.03 ± 0.003a
Available nitrogen (mg/kg)	0.27 ± 0.003a	0.28 ± 0.007a	0.28 ± 0.008a
Organic matter (%)	59.30 ± 0.121b	62.40 ± 0.817c	45.93 ± 0.349a
Ammonium nitrogen (mg/kg)	59.66 ± 1.223a	63.00 ± 0.939ab	63.32 ± 1.810c
pH	7.38 ± 0.298a	7.89 ± 0.500a	8.11 ± 0.241a

“C” represents moving dunes, “SS” represents semi-fixed dunes, and “FS” represents fixed dunes. Different letters in the same row indicate statistically significant differences (*p* < 0.05, one-way ANOVA).

### 3.2 General features of the sequencing data

After quality control, a total of 3,516,828 optimized bacterial sequences were obtained, distributed across the three root zone samples. Specifically, the three successional stages in MUSL contained 2,867,563 optimized sequences in the R samples, 2,535,205 in the F samples, and 2,535,281 in the O samples. The length of the optimized bacterial sequences was primarily 360–380 bp, with an average length of 376 bp. After quality control, a total of 3,936,363 optimized fungal sequences were obtained, which were distributed across the three root zone samples. Specifically, 1,534,082 optimized sequences were identified in the R samples, 1,224,167 in the F samples, and 1,178,114 in the O samples across the three successional stages in MUSL. The length of the optimized fungal sequences was primarily 241–260 bp, with an average length of 245 bp.

The optimized sequences were clustered into OTUs for species classification based on 97% sequence similarity. A total of 7,252 OTUs were generated for bacterial samples, and 1,454 OTUs were generated for fungal samples. The sparse curve of the coverage index approached a saturation plateau as the number of reads increased, indicating that sequencing depth was sufficient for comprehensive data analysis (Supplementary Figure 3).

### 3.3 Diversity and structure of the microbial community

**Figure 1:**
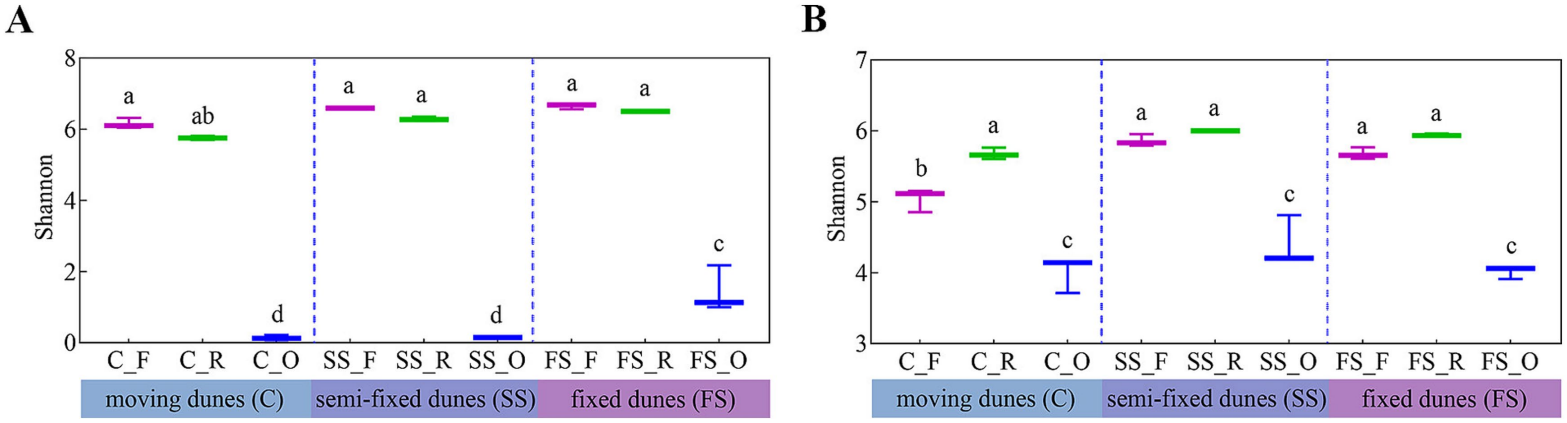
Alpha diversity of microbial communities in the root zone of *Artemisia ordosica* across three successional stages in Mu Us Sandy Land. Shannon indices of **(A)** bacterial and **(B)** fungal communities. Whiskers indicate the lowest and highest values. One-way ANOVAs are used to examine the data, and significant differences at *p* < 0.05 are indicated through different small letters on the bars.

Microbial diversity in each sample was assessed using alpha diversity, measured by the Shannon index (Figure 1). The bacterial Shannon indices were 10.79-fold and 11.29-fold higher in the R and F samples, respectively, than in the O samples (Figure 1A). Similarly, the Shannon index for fungi was 1.42-fold higher in the R samples and 1.33-fold higher in the F samples compared with the O samples (Figure 1B). C samples exhibited the lowest bacterial and fungal diversities across all root zone types (Figure 1).

The influence of soil physical and chemical factors on alpha diversity was analyzed in the root zone of *A. ordosica*. A negative correlation was observed between the Chao1 index and OM content in the bacterial community (*r* = −0.76, *p* = 0.018, linear regression) (Supplementary Figure 4). In the fungal community, the Chao1 index showed significant positive correlations with AK content (*r* = 0.8, *p* = 0.009) and NH_4_^+^-N (*r* = 0.69, *p* = 0.04), as well as a significant negative correlation with TK content (*r* = −0.79, *p* = 0.011) (Supplementary Figure 5).

**Figure 2:**
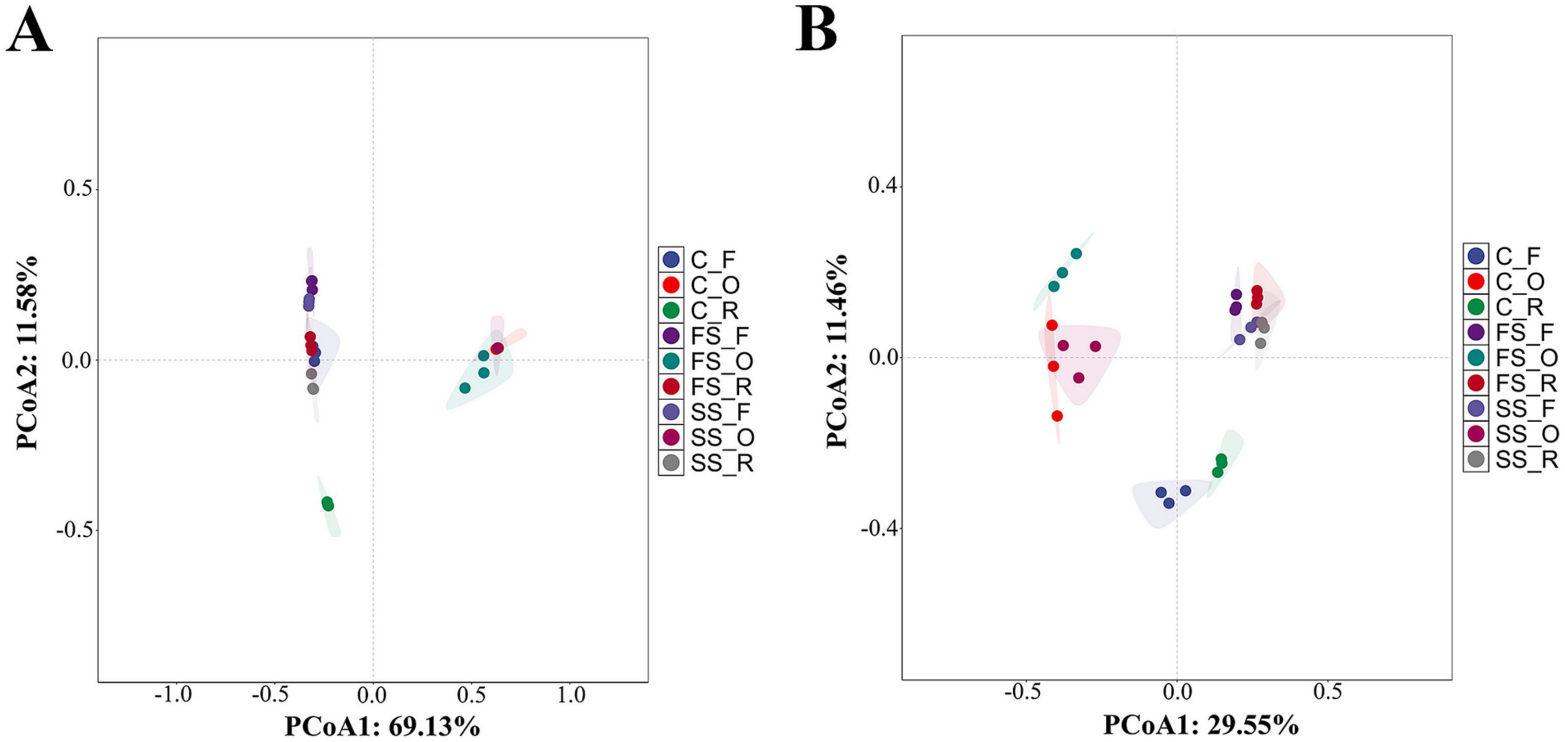
Principal coordinates analysis (PCoA) of microbial communities across the root zones of *Artemisia ordosica* Krasch. at different successional stages in Mu Us Sandy Land [**(A)** bacteria **(B)** fungi]. FS, SS, and C represent fixed dunes, semi-fixed dunes, and moving dunes, respectively. O, F, and R represent root endosphere, non-rhizosphere soil, and rhizosphere soil.

All samples were clearly divided into two groups: endosphere and non-endosphere (Figure 2A). The results of Bray–Curtis dissimilarity-based ADONIS (*R^2^* = 0.927, *p* = 0.001) and ANOSIM (*R^2^* = 0.896, *p* = 0.001) tests confirmed significant differences in bacterial composition across the root zone of *A. ordosica* at different successional stages in MUSL. Additionally, close clustering was observed among root endosphere bacterial communities from C, SS, and FS, indicating high similarity among these communities. However, bacterial communities in C samples were distinct from those in other root zone samples of *A. ordosica*.

Similar to bacterial composition, fungal composition was clearly grouped into endosphere and non-endosphere groups (Figure 2B). The results of Bray–Curtis dissimilarity-based ADONIS (*R^2^* = 0.66, *p* = 0.001) and ANOSIM (*R^2^* = 0.737, *p* = 0.001) tests confirmed significant differences in fungal composition across the root zone of *A. ordosica* at different successional stages in MUSL. Furthermore, clustering was observed among root endosphere fungal communities from C, SS, and FS, particularly between C and SS, suggesting that these fungal communities were highly similar. However, rhizosphere and non-rhizosphere fungal communities of *A. ordosica* in C samples differed from those in the SS and FS samples.

### 3.4 Taxonomic distribution of microbial communities

**Figure 3:**
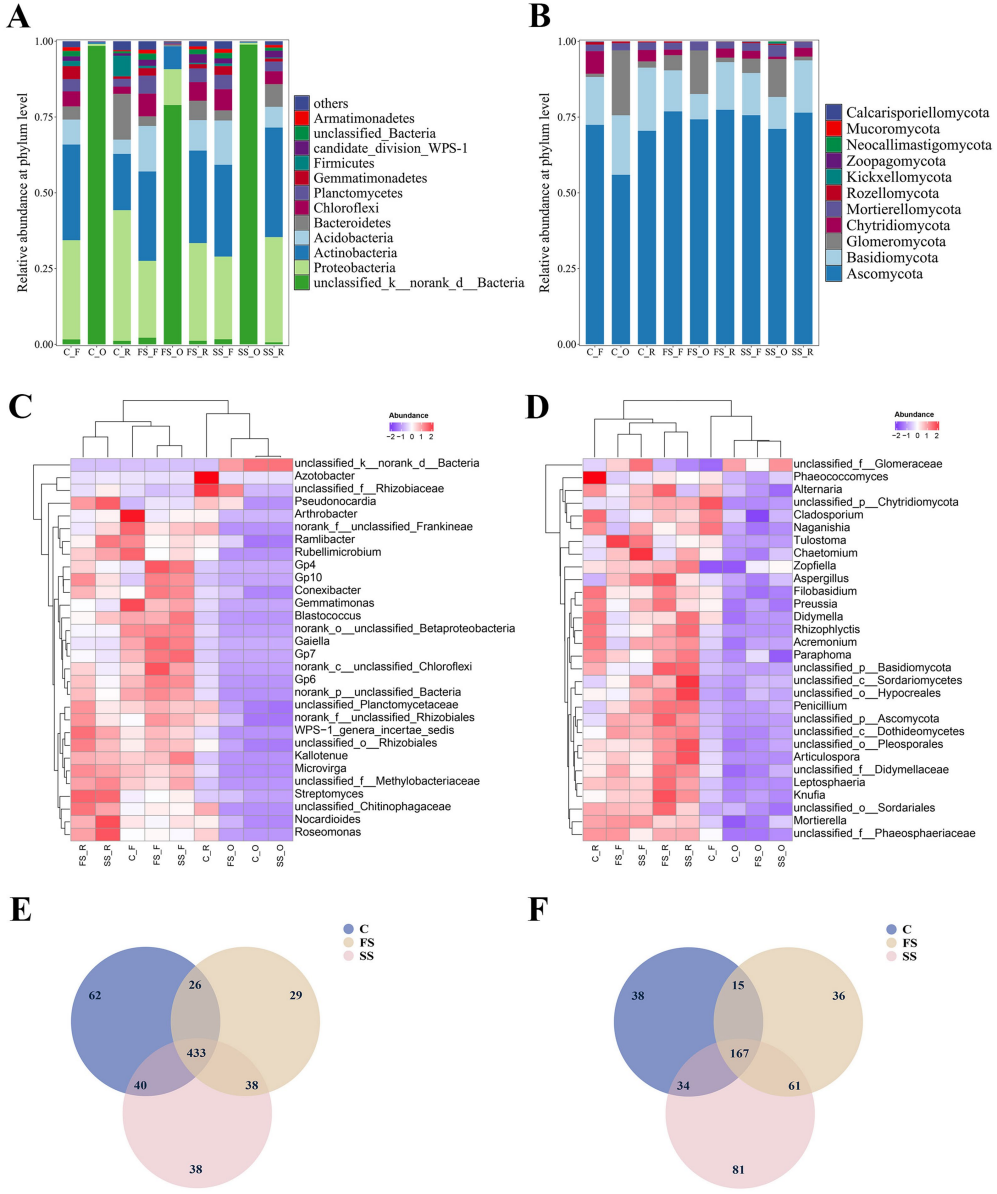
Compositions of the **(A)** bacterial and **(B)** fungal communities at the phylum level in the root zone of *Artemisia ordosica* under various successional stages in Mu Us Sandy Land (MUSL). Heat maps displaying the 30 most abundant **(C)** bacterial and **(D)** fungal genera in the root zone of *A. ordosica*. Color intensity represents the relative abundance of each genus as a percentage of total effective sequences. **(E)** Venn diagram illustrating exclusive and shared bacterial genera across the three successional stages in MUSL. **(F)** Venn diagram illustrating exclusive and shared fungal genera across the three successional stages in MUSL. FS, SS, and C represent fixed dunes, semi-fixed dunes, and moving dunes, respectively. O, F, and R represent root endosphere, non-rhizosphere soil, and rhizosphere soil.

This study identified 3, 9, and 12 major bacterial phyla (i.e., phyla with > 1% relative abundance in at least one group) in the endosphere, rhizosphere, and non-rhizosphere, respectively, of *A. ordosica*. The three most prevalent endosphere bacterial phyla in FS samples were unclassified_k__norank_d__Bacteria (78.89%), Proteobacteria (11.80%), and Actinobacteria (7.57%) (Figure 3A). However, in SS and C samples, nearly all endosphere bacterial sequences (98.87% and 98.45%, respectively) belonged to unclassified_k__norank_d__Bacteria. In the rhizosphere bacterial community, Proteobacteria (32.32%–43.11%) and Actinobacteria (18.58%–36.17%) were the most prevalent phyla, followed by Bacteroidetes (6.38%–15.11%) and Acidobacteria (4.68%–10.06%). Notably, rhizosphere communities in C samples had greater abundances of Proteobacteria, Bacteroidetes, and Firmicutes compared with the other two sampling sites (Figure 3A). The most abundant non-rhizosphere bacterial phyla were Actinobacteria (29.47%–31.56%) and Proteobacteria (25.35%–32.73%), followed by Acidobacteria (8.24%–15.01%) and Chloroflexi (4.98%–7.50%). Compared with the other two sampling sites, Acidobacteria were less common within rhizosphere communities in C samples.

In the fungal community, *A. ordosica* endosphere, rhizosphere, and non-rhizosphere contained four, five, and five dominant phyla (i.e., phyla with >1% relative abundance in at least one group), respectively. As shown in Figure 3B, the four predominant fungal phyla in the endosphere were Ascomycota (55.95%–74.25%), Glomeromycota (12.50%–21.43%), Basidiomycota (8.38%–19.64%), and Mortierellomycota (2.38%–3.91%). The SS samples contained a higher abundance of Mortierellomycota sequences than the other two sampling sites, whereas the FS samples contained a higher abundance of Glomeromycota sequences. In the rhizosphere fungal community, the most abundant phyla were Ascomycota (70.47%–77.42%) and Basidiomycota (15.73%–20.87%), followed by Chytridiomycota (2.89%–3.76%) and Mortierellomycota (1.98%–2.51%). The most abundant phyla in the non-rhizosphere fungal community were Ascomycota (72.44%–76.88%) and Basidiomycota (13.55%–15.81%), followed by Chytridiomycota (1.82%–7.49%) and Glomeromycota (1.07%–5.01%). Notably, non-rhizosphere communities from C samples contained a higher abundance of Chytridiomycota compared with those from the other two sampling sites (Figure 3B).

Hierarchically clustered heat map analysis was performed to identify and evaluate the 30 dominant bacterial genera (Figure 3C). At the genus level, *unclassified_k__norank_d__Bacteria* (92.09%) was the most abundant bacterial taxon in the endosphere of *A. ordosica*. In contrast to the endosphere, *Azotobacter* (3.46%) and *Nocardioides* (3.22%) were dominant bacteria in the rhizosphere of *A. ordosica*, followed by *Microvirga* (2.75%), *Arthrobacter* (2.66%), and *Streptomyces* (2.59%). *Gp6* (4.68%) and *Arthrobacter* (5.16%) were dominant bacteria in the non-endosphere. The bacterial communities of *A. ordosica* were primarily clustered into three categories based on R, F, and O sample types. However, R samples from the moving dunes (C_R) did not cluster with R samples from the other two sampling sites (Figure 3C). Additionally, rhizosphere bacterial communities in SS and FS samples were more closely compared to those in the C samples, similar to findings regarding non-rhizosphere samples (Figure 3C). In contrast, endosphere bacterial communities exhibited greater similarity between C samples and SS samples (Figure 3C).

Moreover, hierarchically clustered heat map analysis was conducted to evaluate the 30 most abundant fungal genera (Figure 3D). Among the endosphere samples, the most abundant fungal genera in *A. ordosica* were *unclassified_f__Glomeraceae* (8.80%), *Paraphoma* (7.61%), and *unclassified_c__Sordariomycetes* (3.21%), followed by *Mortierella* (3.05%) and *Aspergillus* (2.88%). In contrast, fungal communities in the rhizosphere and non-rhizosphere of *A. ordosica* were dominated by *unclassified_p__Ascomycota* (6.48 and 6.58%, respectively) and *unclassified_c__Sordariomycetes* (3.97 and 3.46%, respectively). Compared with the endosphere, these taxa were more abundant in the rhizosphere and non-rhizosphere. Furthermore, heat map analysis indicated that, except for C samples, the fungal communities of *A. ordosica* were primarily clustered into three distinct groups based on R, F, and O sample types (Figure 3D). Fungal communities in SS and FS samples exhibited greater similarity, clustering more closely relative to C samples (Figure 3D).

### 3.5 Venn diagram of microbial communities

In total, 666 bacterial genera were identified in the root zone of *A. ordosica*. Among these, 433 genera were detected in MUSL samples at different successional stages (Figure 3E). C samples contained the greatest number of annotated bacterial genera (126 genera), whereas FS samples contained the fewest (93 genera). Additionally, 62, 38, and 29 genera were unique to the C, SS, and FS samples, respectively (Figure 3E).

In the fungal community, 432 genera were annotated, with 167 genera shared across the three successional stages in MUSL, comprising 38.66% of the total fungal genera. This proportion was lower than that observed for bacteria, suggesting that fungal-specific genera were more characteristic of different successional stages in MUSL. The numbers of unique fungal genera identified in C, SS, and FS samples were 38, 81, and 36, respectively (Figure 3F). The SS plot contained the highest numbers of fungal genera (254) and unique fungal genera (81, corresponding to 18.75%) (Figure 3F).

### 3.6 Relationships between microbial community structure and environmental factors

**Figure 4:**
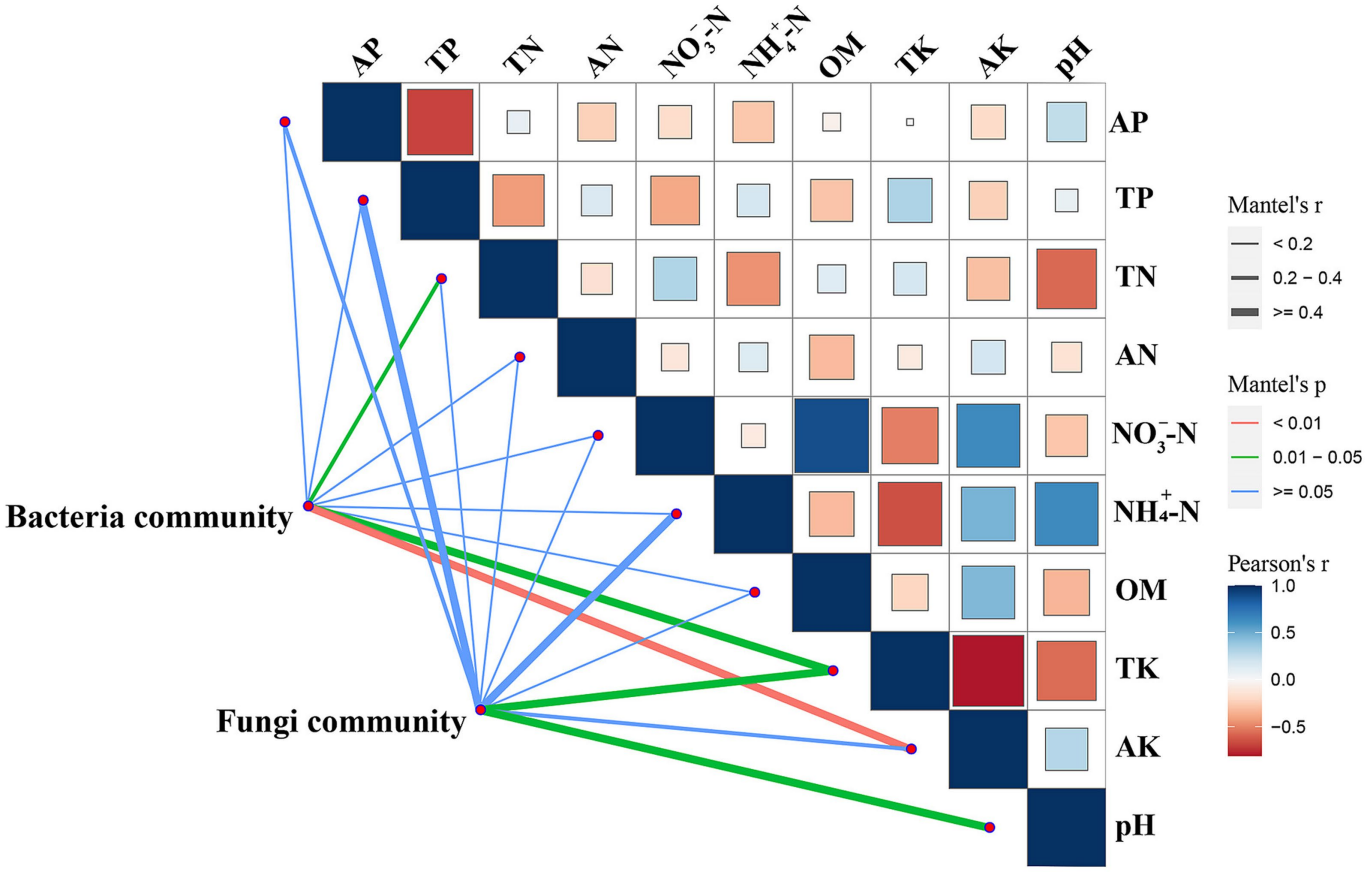
Mantel test heat map analysis of microorganisms in the root zone of *Artemisia ordosica*. The thickness of the lines connecting the microbial community and edaphic physicochemical factors represents Mantel’s *r*, whereas line color indicates Mantel’s *p*. Correlations between two edaphic physicochemical factors are depicted by square size and color; larger squares indicate stronger associations. Pearson’s *r* is reflected by the square color gradient. AP, available phosphorus; TN, total nitrogen; TP, total phosphorus; TK, total potassium; NO_3_^—^N, nitrate nitrogen; AK, available potassium; AN, available nitrogen; NH_4_^+^-N, ammonium nitrogen; OM, organic matter.

Correlations between the microbial community and environmental factors in the root zone of *A. ordosica* were analyzed by Mantel test heat map analysis, as shown in Figure 4. The bacterial community exhibited a strong positive correlation with AK content (*p* < 0.01) and positive correlations with TK and TN contents (both *p* < 0.05). The fungal community also showed positive correlations with TK content and pH (both *p* < 0.05). Additionally, the analysis revealed a negative correlation between TK and AK contents, negative correlations between TN content and both NH_4_^+^-N content and pH, and a positive correlation between OM and NO_3_^−^-N contents. These findings suggest that AK significantly increased the richness of bacterial communities in the root zone of *A. ordosica*, whereas TK promoted the richness of both fungal and bacterial communities.

**Figure 5:**
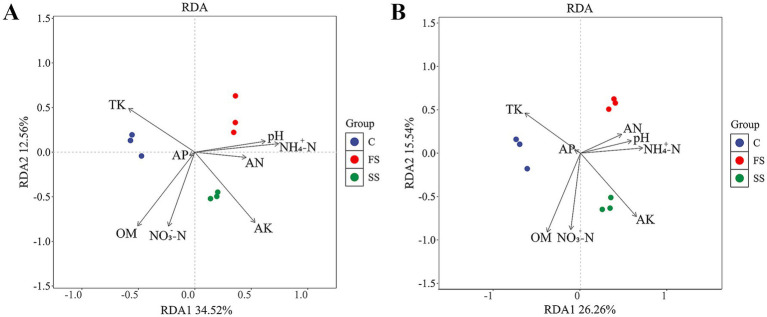
Redundancy analysis (RDA) of correlations between microbial communities and soil physicochemical factors [**(A)** bacteria **(B)** fungi]. Arrows indicate the magnitude and direction of environmental factors influencing bacterial community structure. NH_4_^+^-N, ammonium nitrogen; OM, organic matter; AP, available phosphorus; TP, total phosphorus; TK, total potassium; NO_3_^−^-N, nitrate nitrogen; AK, available potassium; AN, available nitrogen. FS, SS, and C represent fixed dunes, semi-fixed dunes, and moving dunes, respectively.

RDA was performed to assess potential relationships between microbial community composition and environmental parameters. As shown in Figure 5A, the primary soil environmental factors with significant impacts on the composition and assembly of the bacterial community of *A. ordosica* were NO_3_^−^-N (*R*^2^ = 0.8763740, *p* = 0.001), OM (*R*^2^ = 0.9850710, *p* = 0.003), AK (*R*^2^ = 0.9605907, *p* = 0.004) and TK (*R*^2^ = 0.6475219, *p* = 0.042). The analysis also indicated that the bacterial community structure of the FS samples was positively correlated with pH but negatively correlated with OM and NO_3_^−^-N contents. The bacterial community structure of SS samples showed a strong positive correlation with AK but was negatively correlated with TK content. However, the bacterial community composition in C samples was negatively correlated with NH_4_^+^-N content and pH.

The primary soil environmental parameters that significantly influenced the composition and assembly of the fungal community of *A. ordosica* were OM (*R*^2^ = 0.98765748, *p* = 0.002), NO_3_^−^-N (*R*^2^ = 0.78070967, *p* = 0.008), AK (*R*^2^ = 0.93774130, *p* = 0.009), and TK (*R*^2^ = 0.62823881, *p* = 0.049), as shown in Figure 5B. Additionally, AK content was positively correlated with the fungal community structure of SS samples (Figure 5B).

### 3.7 Molecular ecological network of microbial communities

**Figure 6:**
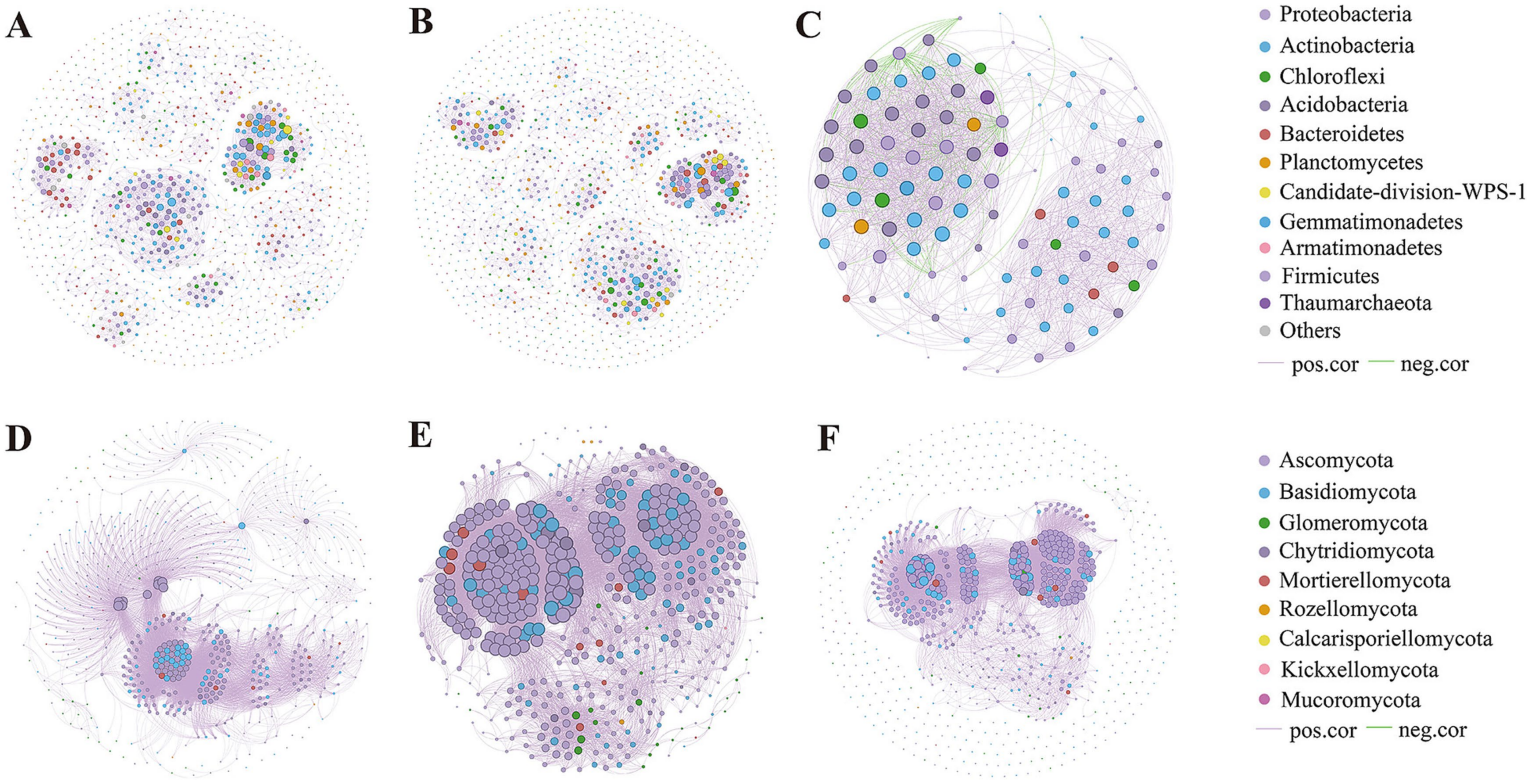
Bacterial **(A,B,C)** and fungal **(D,E,F)** networks of dominant microbiota at the phylum level in moving dunes (C) **(A,D)**, semi-fixed dunes (SS) **(B,E)**, and fixed dunes (FS) **(C,F)**. OTU abundance is represented by node size, and the phylum-level taxonomic classification is indicated by node color. Positive correlations (purple) and negative correlations (green) are represented by the edge color.

To examine correlations among root zone microorganisms in *A. ordosica* at different successional stages in MUSL, a network diagram was constructed depicting microbial co-occurrence patterns at each stage. In the bacterial co-occurrence network analysis, most nodes exhibited positive interactions, whereas in the fungal network, nearly all nodes demonstrated positive associations, suggesting that microbial communities in the root zone of *A. ordosica* formed a highly interconnected network through synergistic relationships during succession (Figure 6). For bacterial communities, the number of connections varied across sites: 4,001 connections in C, 3,497 in SS, and 1,739 in FS. These findings suggest that moving dunes (C) had a more complex bacterial network than the other sites. The modularity of bacterial networks in C and SS samples was higher than in FS samples, indicating that C and SS exhibited greater network complexity and a more defined functional organization. The average degree of the fungal community was relatively high, suggesting strong species interactions. Additionally, the modularities of fungal communities in C and FS were > 0.4, indicating that these sites maintained modular structures.

### 3.8 Culturomics insight of microbial community with different isolation methods

To characterize the culturable microbiota associated with *A. ordosica*, conventional streak plate and colony pick methods were employed for isolation of microorganisms across all samples. In parallel, a high-throughput cultivation method specifically designed for bacterial isolation was implemented to expand the taxonomic coverage of culturable bacteria.

**Figure 7:**
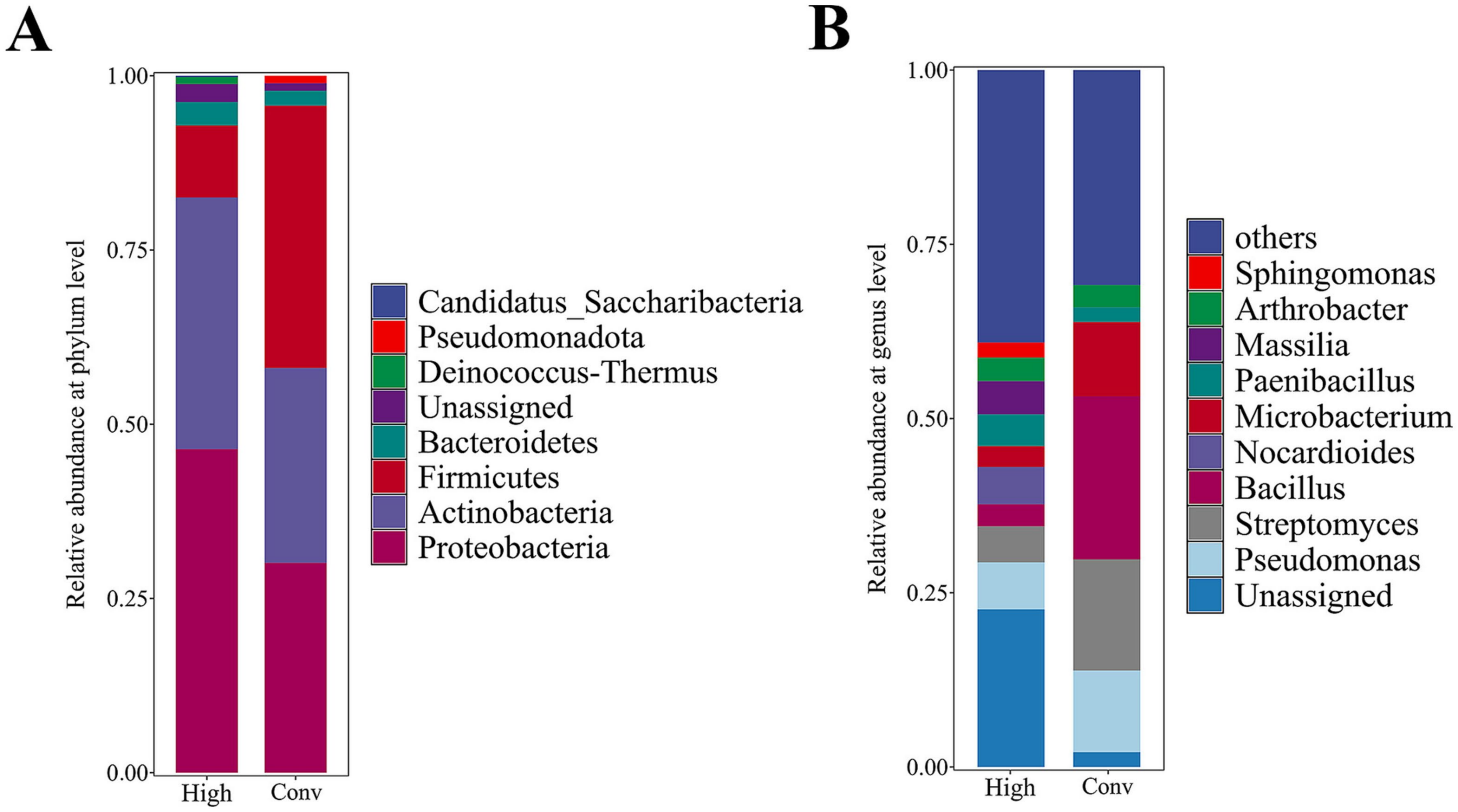
Taxonomic composition of bacteria in the root zone of *Artemisia ordosica* analyzed using the conventional streak plate and colony pick methods (Conv) and the high-throughput cultivation and identification method (High). **(A)** Composition of bacterial communities at the phylum level obtained using the two culture methods. **(B)** Composition of bacterial communities at the genus level obtained using the two culture methods. The 10 genera with the highest relative abundance are shown; the remaining genera are combined into the “Other” category.

The conventional streak plate and colony pick methods yielded 93 purified bacterial strains which belonged to six phyla, 30 genera. The core bacterial phyla captured by this method were Firmicutes (relative abundance: 37.63%), Proteobacteria (relative abundance: 30.11%), Actinobacteria (27.96%), and Bacteroidetes (2.15%) (Figure 7A). In addition, *Bacillus* (23.66%) was the most predominant bacterial genus, followed by *Streptomyces* (16.13%) and *Pseudomonas* (11.83%) (Figure 7B). Meanwhile, a total of 14 fungal strains were successfully isolated by colony picking. Taxonomic classification based on ITS-rDNA sequencing revealed that the isolates were predominantly composed of the genus *Penicillium* (relative abundance: 35.71%), followed by *Aspergillus* (21.43%) and *Cladosporium* (14.29%).

The high-throughput cultivation and identification method, coupled with amplicon sequence variant (ASV) analysis, which identified 504 unique ASVs. Saturation analysis of sequencing-derived strains demonstrated gradual stabilization of diversity metrics (Supplementary Figure 6), confirming that our 48 × 96-well plate cultivation system provided sufficient strain recovery. Colony purity assessment revealed that 17.7% of wells contained >95% pure cultures (Supplementary Figure 7). The high-throughput method captured seven phyla, 102 genera bacterial strains. The most abundant phyla isolated using this method were Proteobacteria (relative abundance: 46.43%), Actinobacteria (relative abundance: 36.11%), Firmicutes (10.32%), and Bacteroidetes (3.37%) (Figure 7A). At the genus level, *Unassigned* (22.62%) and *Pseudomonas* (6.75%) were dominant bacteria, followed by *Nocardioides* (5.36%), *Streptomyces* (5.16%) (Figure 7B). Intriguingly, four strains belonging to the *Deinococcus* genera (phylum Deinococcus-Thermus) were isolated via this approach, potentially linked to the adaptation of *A. ordosica* to arid sandy environments.

## 4 Discussion

### 4.1 Differences in microbial community structure and diversity across root zones and successional stages in sandy land

Microorganisms inhabiting the plant endosphere and rhizosphere serve as bridges among plants, microbial communities, and the surrounding environment (Niu et al., 2017). Our findings demonstrated that microbial diversity was lowest in moving dunes (C), consistent with their sparse vegetation coverage (Supplementary Figure 1). Considering both microbial diversity patterns and geomorphic characteristics of moving dunes, it is likely that scarce vegetation resources contributed to low microbial diversity. This finding is consistent with previous research indicating that plants promote the growth of soil bacteria and fungi during community succession (Li et al., 2013).

Notably, beta diversity patterns revealed divergent community structures across successional stages, with C samples exhibiting the most pronounced differences in rhizosphere and non-rhizosphere compartments (Figure 2). However, root endosphere communities maintained high structural similarity regardless of site, reinforcing their documented stability against environmental variations (Edwards et al., 2015; Wagner et al., 2016). Environmental differences among various plant compartments likely contribute to the stability and limited diversity of the root endosphere. Additionally, plant immunity serves as a selective barrier, limiting microbial entry and colonization, thereby maintaining the root endosphere as a relatively stable and less diverse environment (de Lamo et al., 2021; Oukala et al., 2021). Compared with the simpler environment of the endosphere, the rhizosphere receives nutrients and signaling molecules secreted by plants, which recruit specific microorganisms and promote their colonization (Jin et al., 2019; Dhungana et al., 2023). Complex physical and chemical interactions occur in the rhizosphere during plant–microbe interactions (Liu et al., 2020). These interactions require support from multiple functional microorganisms, leading to the formation of a complex and diverse rhizosphere microbial community (Kuppe et al., 2022). In contrast, the non-rhizosphere soil environment lacks plant-derived secretions, resulting in a microbial community structure distinct from that of the rhizosphere.

### 4.2 Factors influencing microbial community diversity and composition

The diversity and composition of microbial communities are strongly influenced by soil physicochemical properties, including nutrient availability and pH (Wang et al., 2024; Eduah et al., 2024). Consistent with previous research, our findings highlight nitrogen and phosphorus—essential for bacterial metabolism—as major determinants of microbial community dynamics (Liao et al., 2015). Soil pH is also a key determinant of the soil microbiome (Zhang et al., 2022; Liu et al., 2023). Interestingly, bacterial communities in moving dunes exhibited a negative correlation with pH, suggesting niche-specific adaptations to alkaline conditions. Microorganisms acquire nutrients by decomposing OM, and microbial community composition can be strongly influenced by altered organic material availability (Fagervold et al., 2014; Yang et al., 2024). This mechanistic link explains the strong association observed between OM content and both bacterial and fungal communities in our redundancy analysis.

Co-occurrence networks provide insights into microbial interactions and community formation (Williams et al., 2014). The higher connectivity in moving dunes (C) implies that early successional stages foster more intricate microbial networks, possibly due to greater environmental stress or niche opportunities. The pronounced modularity in bacterial networks at C and SS sites represents a higher degree of network complexity and suggests that network modules exhibited clearer structural organization and functional differentiation. This modular structure likely enhanced the functional efficiency of microbial interactions (Supplementary Table 2). Notably, most nodes exhibited positive interactions, particularly among fungal communities, which indicates that microbial communities in the root zone of *A. ordosica* formed a highly interconnected network, facilitated by synergistic interactions among species during succession in MUSL (Figure 6).

### 4.3 Dominant genera of microorganisms in the root zone

At the genus level, several functionally important taxa emerged as key components of the *A. ordosica* root zone microbiome. The rhizosphere was particularly enriched with *Arthrobacter*, *Nocardioides* and *Streptomyces*, bacterial genera with plant growth-promoting capabilities and stress tolerance traits. Genome sequence analysis of *Arthrobacter koreensis* 5J12A revealed that *Arthrobacter* spp. can tolerate desiccation and promote plant growth (Manzanera et al., 2015). The novel desert-adapted strain KC13(T), isolated from the Karakum Desert, was identified as *Nocardioides* spp. (Saygin et al., 2024). *Streptomyces* spp. also exhibits growth-promoting effects on plants. Strain Z38, isolated from contaminated root tissue, was confirmed to enhance plant growth (Costa et al., 2020). In greenhouse experiments, seed treatments with *Streptomyces* TKSC3 and *Streptomyces* SS8 led to increases of 20%, 23%, and 33% in germination rate, root length, and dry weight, respectively. Additionally, single and combined *Streptomyces* treatments successfully inhibited rice leaf blight (Hata et al., 2021). These findings suggest that representative strains of these genera, which are enriched in the root zone of *A. ordosica*, contribute to plant growth promotion and drought resistance. Similarly, fungal communities featured *Paraphoma* as a dominant endophytic genus, which has been demonstrated to possess the ability to enhance plant growth and secondary metabolite production under abiotic stress conditions (Li et al., 2024). These findings highlight the roles of dominant genera, such as *Paraphoma*, in plant growth promotion and their potential applications in phytoremediation strategies.

### 4.4 Analysis of microbial taxonomic composition based on different isolation methods

The cultivation approaches employed in this study successfully captured diverse bacterial and fungal communities associated with *A. ordosica* roots, revealing distinct taxonomic profiles through different isolation methods. The conventional streak plate and colony pick methods predominantly recovered Firmicutes, particularly *Bacillus* species, which may reflect their fast-growing nature and spore-forming capabilities under standard culture conditions. The fungal isolates were dominated by *Penicillium* and *Aspergillus*, genera well-known for their stress tolerance and ecological versatility in harsh environments (Yovchevska et al., 2025; Ortega-Villaizán et al., 2025; Haseena et al., 2024). These findings collectively expand our understanding of the culturable microbiome associated with *A. ordosica*, providing a valuable repository of microbial resources for further investigation.

The high-throughput cultivation method demonstrated enhanced capacity to isolate rare and slow-growing taxa, as evidenced by the recovery of *Deinococcus* species—radiation-resistant extremophiles particularly suited to arid environments (Rainey et al., 2005). The limitations of laboratory-based microbial culture are primarily due to difficulties in replicating environmental conditions and microbial competition. Cultivation in 96-well plates helps mitigate these challenges by diluting the medium, thus facilitating the growth of rare and slow-growing bacteria (Lewis et al., 2021).

The isolated strains, particularly those belonging to drought-adapted genera like *Deinococcus* and *Streptomyces*, represent promising candidates for developing microbial inoculants to enhance plant stress resistance. Future studies should first validate their phytobeneficial effects through controlled pot experiments under water-deficit conditions. Subsequently, integrated transcriptomic and metabolomic analyses could elucidate the molecular mechanisms underlying their plant growth promotion and drought mitigation capabilities. Finally, the potential of these candidate strains to serve as microbial inoculants for sustainable agriculture in arid regions warrants exploration.

## 5 Conclusion

This study characterized the microbial community profiles of the root zone (i.e., rhizosphere soil, non-rhizosphere soil, and root endosphere) of *A. ordosica* at different successional stages (i.e., moving dunes, semi-fixed dunes, and fixed dunes) in MUSL, China, using integrated metagenomic and culturomic approaches. Notably, the Shannon indices for bacteria in the rhizosphere soil and non-rhizosphere soil were 10.79-fold and 11.29-fold higher, respectively, compared with the root endosphere. Furthermore, across all root zone types, bacterial and fungal diversities were lowest in samples from moving dunes. PCoA analysis was implemented to reveal distinct microbial community structures across successional stages, such that the root endosphere substantially differed from other zones. Environmental factors, including NO_3_^−^-N, OM, AK, and TK contents, significantly influenced microbial community composition. Among the dominant taxa, genera such as *Arthrobacter* and *Paraphoma* were identified, suggesting potential roles in supporting *A. ordosica* growth. Meanwhile, in the bacterial and fungal co-occurrence network analysis, most nodes exhibited positive interactions, suggesting that microbial communities in the root zone of *A. ordosica* formed a highly interconnected network through synergistic relationships during succession.

Conventional streak plate and colony pick methods yielded 93 bacterial isolates (six phyla, 30 genera) dominated by Firmicutes (37.63%) and *Bacillus* (23.66%), alongside 14 fungal strains primarily comprising *Penicillium* (35.71%) and *Aspergillus* (21.43%)—taxa with documented stress-tolerance in arid ecosystems. Besides, the high-throughput method captured a broader culturable bacterial diversity, isolating 504 unique ASVs across seven phyla and 102 genera, including Proteobacteria assemblages (46.43%) and rare extremophiles (Deinococcus-Thermus). The high-throughput cultivation protocol substantially expanded the recoverable microbial diversity, addressing the “great plate count anomaly” in sandy land soils.

These findings provide metagenomic with culturomics insights into microbial communities in the root zone of *A. ordosica* at different successional stages in Mu Us Sandy Land that may contribute to desertification mitigation. Characterization of the root-associated microbial community, particularly the enrichment of *Arthrobacter* species, identified candidate taxa for bioinoculant development aimed at enhancing pioneer plant establishment through rhizosphere microbiome engineering. This study effectively bridges microbial ecological theory and practical bioremediation, offering a pathway for the targeted use of sandy land-adapted microbial resources.

## Data Availability

The original contributions presented in the study are publicly available. This data can be found at: https://www.ncbi.nlm.nih.gov/, accession numbers PRJNA1244809, PRJNA1246532, and PRJNA1244737.

## Footnotes

^1^ http://www.r-project.org/

^2^ https://www.cloudtutu.com

^3^ https://www.ezbiocloud.net/
