# Supplementary material for: Root zone microbial communities of Artemisia ordosica Krasch. at different successional stages in Mu US Sandy Land: a metagenomic perspective with culturomics insights
Source: Front Microbiol. 2025 May 9;16:1585700. doi: 10.3389/fmicb.2025.1585700 (PMC12098380; doi:10.3389/fmicb.2025.1585700)
Supplement: Supplementary file 1 [file Table_1.docx]

***Supplementary Material***

1. **Supplementary Figures and Tables**
   1. **Supplementary Tables**

**Supplementary Table 1** Complete list of GenBank accession numbers

| **Submission ID** | **Accession number** |
| --- | --- |
| SUB1522736816S-1-3.23739322 | PV428570 |
| SUB1522736816S-1-2.23739320 | PV428571 |
| SUB1522736816S-1-1.23739318 | PV428572 |
| SUB1522736816S-1.24018240 | PV428573 |
| SUB1522736816S-1.23720155 | PV428574 |
| SUB152273680015_32823042100273_(10) | PV428575 |
| SUB152273680013_32823040700350_(7) | PV428576 |
| SUB152273680011_32823042100271_(22) | PV428577 |
| SUB152273680009_32823051900420_(CR2-8) | PV428578 |
| SUB152273680009_32823042100270_(7) | PV428579 |
| SUB152273680007_32823051900419_(CR2-13) | PV428580 |
| SUB152273680007_32823042100269_(14) | PV428581 |
| SUB152273680005_32823042600412_(35) | PV428582 |
| SUB152273680005_32823042100268_(15) | PV428583 |
| SUB152273680001_32823051900416_(SS-O-3) | PV428584 |
| SUB152273680001_32823042100266_(8) | PV428585 |
| SUB15227368SSR2-25.21255310 | PV428586 |
| SUB15227368SSR2-24.21255308 | PV428587 |
| SUB15227368SSR2-23.21255306 | PV428588 |
| SUB15227368SSR2-22.21255304 | PV428589 |
| SUB15227368SSR2-20.21255300.seq | PV428590 |
| SUB15227368SSR2-19.21255298 | PV428591 |
| SUB15227368SSR2-17.21179420 | PV428592 |
| SUB15227368SSR2-14.21179414 | PV428593 |
| SUB15227368SSR2-13.21179412 | PV428594 |
| SUB15227368SSR2-10.21179406 | PV428595 |
| SUB15227368SSR1-3.21179394 | PV428596 |
| SUB15227368SSR1-2.21179392 | PV428597 |
| SUB15227368SSR1-1.21179390 | PV428598 |
| SUB15227368SSF2-15.21255318 | PV428599 |
| SUB15227368SSF2-13.21255314 | PV428600 |
| SUB15227368SSF2-12.21255312.seq | PV428601 |
| SUB15227368SSF2-11.21179442 | PV428602 |
| SUB15227368SSF2-10.21179440 | PV428603 |
| SUB15227368SSF2-9.21179404 | PV428604 |
| SUB15227368SSF2-6.21179432 | PV428605 |
| SUB15227368SSF2-5.21179430 | PV428606 |
| SUB15227368SSF2-4.21179428 | PV428607 |
| SUB15227368SSF1-12.21255344 | PV428608 |
| SUB15227368SSF1-9.21255338 | PV428609 |
| SUB15227368SSF1-8.21255336 | PV428610 |
| SUB15227368SSF1-7.21255334 | PV428611 |
| SUB15227368SSF1-6.21255332 | PV428612 |
| SUB15227368SSF1-4.21255328 | PV428613 |
| SUB15227368SSF1-3.21255326 | PV428614 |
| SUB15227368SSF1-2.21255324 | PV428615 |
| SUB15227368FSR2-7.21179396 | PV428616 |
| SUB15227368FSF2-2.21179400 | PV428617 |
| SUB15227368FSF2-1.21179398 | PV428618 |
| SUB152273680055_32823042600437_(6) | PV428619 |
| SUB152273680051 | PV428620 |
| SUB152273680049_32823051900440_(CR1-2) | PV428621 |
| SUB152273680047_32823051900439_(SS-O-1) | PV428622 |
| SUB152273680045_32823051900438_(SS-O-4) | PV428623 |
| SUB152273680043_32823051900437_(CR2-7) | PV428624 |
| SUB152273680043_32823042600431_(41) | PV428625 |
| SUB152273680041_32823051900436_(CR2-6) | PV428626 |
| SUB152273680039_32823042600429_(23) | PV428627 |
| SUB152273680039_32823042100285_(3) | PV428628 |
| SUB152273680037_32823042100284_(19) | PV428629 |
| SUB152273680035_32823051900433_(CR2-5) | PV428630 |
| SUB152273680035_32823042100283_(11) | PV428631 |
| SUB152273680033_32823051900432_(CR1-3) | PV428632 |
| SUB152273680033_32823042600426_(42) | PV428633 |
| SUB152273680033_32823042100282_(18) | PV428634 |
| SUB152273680031_32823042100281_(9) | PV428635 |
| SUB152273680029_32823042100280_(2) | PV428636 |
| SUB152273680027_32823051900429_(CR2-3) | PV428637 |
| SUB152273680027_32823042100279_(12) | PV428638 |
| SUB152273680025_32823051900428_(CR2-1) | PV428639 |
| SUB152273680025_32823042600422_(36) | PV428640 |
| SUB152273680025_32823042100278_(17) | PV428641 |
| SUB15227368(CR1-5) | PV428642 |
| SUB152273680023_32823042600421_(26) | PV428643 |
| SUB152273680023_32823042100277_(21) | PV428644 |
| SUB152273680023_32823040700355_(12) | PV428645 |
| SUB152273680021_32823040700354_(11) | PV428646 |
| SUB152273680019_32823051900425_(CR1-1) | PV428647 |
| SUB152273680019_32823042100275_(20) | PV428648 |
| SUB152273680017_32823051900424_(CR2-9) | PV428649 |
| SUB152273680017_32823042600418_(22) | PV428650 |
| SUB152273680017_32823042100274_(4) | PV428651 |
| SUB1522736816S-SS-R16.22824072 | PV428652 |
| SUB1522736816S-SS-R15.22824074 | PV428653 |
| SUB1522736816S-SS-R5.22864674 | PV428654 |
| SUB1522736816S-SS-R4.22864650 | PV428655 |
| SUB1522736816S-SS-R-3-5.23700733 | PV428656 |
| SUB1522736816S-SS-R-3-4.23700794 | PV428657 |
| SUB1522736816S-SS-R-3-3.23047884 | PV428658 |
| SUB1522736816S-SS-R-3-2.23700735 | PV428659 |
| SUB1522736816S-SS-R-3-1.23047882 | PV428660 |
| SUB1522736816S-SS-R3.22864648 | PV428661 |
| SUB1522736816S-SS-R2-29.21355216 | PV428662 |
| SUB1522736816S-SS-R2-28.21355218 | PV428663 |
| SUB1522736816S-SS-R2-27.21355220 | PV428664 |
| SUB1522736816S-SS-R2-2.22511721 | PV428665 |
| SUB1522736816S-SS-R2-1.23047886 | PV428666 |
| SUB1522736816S-SS-R2.22864646 | PV428667 |
| SUB1522736816S-SS-R1-14.22640243 | PV428668 |
| SUB1522736816S-SS-R1-13.22640241 | PV428669 |
| SUB1522736816S-SS-R1-11.22640237 | PV428670 |
| SUB1522736816S-SS-R1-10.22640235 | PV428671 |
| SUB1522736816S-SS-R1-10.21355252 | PV428672 |
| SUB1522736816S-SS-R1-9.22640233 | PV428673 |
| SUB1522736816S-SS-R1-9.21355254 | PV428674 |
| SUB1522736816S-SS-R1-8.21355256 | PV428675 |
| SUB1522736816S-SS-R1-7.22640247 | PV428676 |
| SUB1522736816S-SS-R1-7.21355258 | PV428677 |
| SUB1522736816S-SS-R1-6.22824082 | PV428678 |
| SUB1522736816S-SS-R1-6.22511719 | PV428679 |
| SUB1522736816S-SS-R1-5.22954790 | PV428680 |
| SUB1522736816S-SS-R1-5.22511717 | PV428681 |
| SUB1522736816S-SS-R1-4.22511715 | PV428682 |
| SUB1522736816S-SS-R1-3.22511713 | PV428683 |
| SUB1522736816S-SS-R1-2.22511711 | PV428684 |
| SUB1522736816S-SS-R1-1.23047912 | PV428685 |
| SUB1522736816S-SS-R1-1.22511709 | PV428686 |
| SUB1522736816S-SS-R1.22864644 | PV428687 |
| SUB1522736816S-SS-O-3-11.22824064 | PV428688 |
| SUB1522736816S-SS-O-3-10.22824056 | PV428689 |
| SUB1522736816S-SS-O-3-8.22824060 | PV428690 |
| SUB1522736816S-SS-O-3-6.22954772 | PV428691 |
| SUB1522736816S-SS-O-3-5.23103273 | PV428692 |
| SUB1522736816S-SS-O-3-4.23047904 | PV428693 |
| SUB1522736816S-SSO.24018230 | PV428694 |
| SUB1522736816S-SS-F2-2.22954770 | PV428695 |
| SUB1522736816S-SS-F2-1.22954774 | PV428696 |
| SUB1522736816S-SS-F1-17.21355260 | PV428697 |
| SUB1522736816S-SS-F1-16.21355262 | PV428698 |
| SUB1522736816S-SS-F1-14.21355266 | PV428699 |
| SUB1522736816S-R2-SS-26.22824084 | PV428700 |
| SUB1522736816S-FS-R2-33.21548445 | PV428701 |
| SUB1522736816S-FS-R2-32.21548441 | PV428702 |
| SUB1522736816S-FS-R2-31.21548467 | PV428703 |
| SUB1522736816S-FS-R2-29.21548409 | PV428704 |
| SUB1522736816S-FS-R2-28.21548455 | PV428705 |
| SUB1522736816S-FS-R2-26.21548411 | PV428706 |
| SUB1522736816S-FS-R2-25.21548413 | PV428707 |
| SUB1522736816S-FS-R2-24.21548463 | PV428708 |
| SUB1522736816S-FS-R2-23.21548415 | PV428709 |
| SUB1522736816S-FS-R2-21.21355280 | PV428710 |
| SUB1522736816S-FS-R2-20.21355274 | PV428711 |
| SUB1522736816S-FS-R2-19.21355276 | PV428712 |
| SUB1522736816S-FS-R2-17.21355278 | PV428713 |
| SUB1522736816S-FS-R2-16.21355272 | PV428714 |
| SUB1522736816S-FS-R2-15.21355222 | PV428715 |
| SUB1522736816S-FS-R2-14.21355224 | PV428716 |
| SUB1522736816S-FS-R2-13.21355226 | PV428717 |
| SUB1522736816S-FS-R2-12.21355228 | PV428718 |
| SUB1522736816S-FS-R2-11.22824080 | PV428719 |
| SUB1522736816S-FS-R2-11.21355230 | PV428720 |
| SUB1522736816S-FSR2-10.22824094 | PV428721 |
| SUB1522736816S-FS-R2-10.21355232 | PV428722 |
| SUB1522736816S-FS-R2-9.22954776 | PV428723 |
| SUB1522736816S-FSR2-9.22824092 | PV428724 |
| SUB1522736816S-FSR2-8.22824090 | PV428725 |
| SUB1522736816S-FS-R1-16.21548453 | PV428726 |
| SUB1522736816S-FS-R1-15.21548459 | PV428727 |
| SUB1522736816S-FS-R1-14.21548443 | PV428728 |
| SUB1522736816S-FS-R1-13.21548457 | PV428729 |
| SUB1522736816S-FS-O-14.22864672 | PV428730 |
| SUB1522736816S-FS-O-13.22864670 | PV428731 |
| SUB1522736816S-FS-O-12-1.23047888 | PV428732 |
| SUB1522736816S-FS-O-11.22864666 | PV428733 |
| SUB1522736816S-FS-O-10.22864664 | PV428734 |
| SUB1522736816S-FS-O-9-1.23103281 | PV428735 |
| SUB1522736816S-FS-O-9.23047918 | PV428736 |
| SUB1522736816S-FS-O-8.22864660 | PV428737 |
| SUB1522736816S-FS-O-7.22864658 | PV428738 |
| SUB1522736816S-FS-O-6-1.23047896 | PV428739 |
| SUB1522736816S-FS-O-6.23047894 | PV428740 |
| SUB1522736816S-FS-O-5-2.23103269 | PV428741 |
| SUB1522736816S-FS-O-5.23047890 | PV428742 |
| SUB1522736816S-FS-O-5.22640245 | PV428743 |
| SUB1522736816S-FS-O-4.23047906 | PV428744 |
| SUB1522736816S-FS-O-4.22640249 | PV428745 |
| SUB1522736816S-FS-O-3-3.23103267 | PV428746 |
| SUB1522736816S-FS-O-3-2.23103297 | PV428747 |
| SUB1522736816S-FS-O-3-2.22824068 | PV428748 |
| SUB1522736816S-FS-O-3.23047892 | PV428749 |
| SUB1522736816S-FS-O-3.22640251 | PV428750 |
| SUB1522736816S-FS-O-2.22864654 | PV428751 |
| SUB1522736816S-FS-O-2.22640253 | PV428752 |
| SUB1522736816S-FS-O-1.22864652 | PV428753 |
| SUB1522736816S-FS-O-1.22640255 | PV428754 |
| SUB1522736816S-FS-F2-11.23700796 | PV428755 |
| SUB1522736816S-FS-F2-8.21548469 | PV428756 |
| SUB1522736816S-FS-F2-7.21548431 | PV428757 |
| SUB1522736816S-FS-F2-6.21548433 | PV428758 |
| SUB1522736816S-FS-F2-4.21548421 | PV428759 |
| SUB1522736816S-FS-F2-3.21548437 | PV428760 |
| SUB1522736816S-FS-F2-2-1.21548451 | PV428761 |
| SUB1522736816S-FS-F2-2.21548439 | PV428762 |
| SUB1522736816S-FSF2-1.23700727 | PV428763 |
| SUB1522736816S-FS-F2-1.22511723 | PV428764 |
| SUB1522736816S-FS-F2-1.21355282 | PV428765 |
| SUB1522736816S-FS-F1-9.21355250 | PV428766 |
| SUB1522736816S-FS-F1-6.21355244 | PV428767 |
| SUB1522736816S-FS-F1-4.21355240 | PV428768 |
| SUB1522736816S-FS-F1-1.21355234 | PV428769 |
| SUB1522736816S-F1SS-1.22849541 | PV428770 |
| SUB1522736816S-CR2-5.23700729 | PV428771 |
| SUB1522736816S-CR2-4.23700737 | PV428772 |
| SUB1522736816S-CR2-3.23700741 | PV428773 |
| SUB1522736816S-CR2-2.24018234 | PV428774 |
| SUB1522736816S-CR2-2.23700745 | PV428775 |
| SUB1522736816S-CR2.22824066 | PV428776 |
| SUB1522736816S-CR1-30.23700784 | PV428777 |
| SUB1522736816S-CR1-16.23700751 | PV428778 |
| SUB1522736816S-CR1-8.23700731 | PV428779 |
| SUB1522736816S-CR1-1-3.23700723 | PV428780 |
| SUB1522736816S-CR1-1-2.23700725 | PV428781 |
| SUB1522736816S-CR1.24018236 | PV428782 |
| SUB1522736816S-CR1.22824070 | PV428783 |
| SUB1522736816S-C-O-T14.23163693 | PV428784 |
| SUB1522736816S-C-O-T13.24018232 | PV428785 |
| SUB1522736816S-C-O-T8.23163691 | PV428786 |
| SUB1522736816S-C-O-T5-1.23700790 | PV428787 |
| SUB1522736816S-C-O-T2-1.23700788 | PV428788 |
| SUB1522736816S-C-O-SHUI.23103291 | PV428789 |
| SUB1522736816S-C-O-R18.23163677 | PV428790 |
| SUB1522736816S-C-O-R8-1.23163675 | PV428791 |
| SUB1522736816S-C-O-R8.23163667 | PV428792 |
| SUB1522736816S-C-O-R2.23700739 | PV428793 |
| SUB1522736816S-C-O-R1.23163687 | PV428794 |
| SUB1522736816S-C-O-GEN.23103293 | PV428795 |
| SUB1522736816S-C-O-24.23163701 | PV428796 |
| SUB1522736816S-C-O-23.23163697 | PV428797 |
| SUB1522736816S-C-O-22.23700743 | PV428798 |
| SUB1522736816S-C-O-21.23163695 | PV428799 |
| SUB1522736816S-C-O-20-1.23163669 | PV428800 |
| SUB1522736816S-C-O-19-1.23700792 | PV428801 |
| SUB1522736816S-C-O-19.23700798 | PV428802 |
| SUB1522736816S-C-O-16.23163673 | PV428803 |
| SUB1522736816S-C-O-14-1.23163663 | PV428804 |
| SUB1522736816S-C-O-14.23163671 | PV428805 |
| SUB1522736816S-C-O-13.23163665 | PV428806 |
| SUB1522736816S-C-O-4-GEN.23103279 | PV428807 |
| SUB1522736816S-C-O-3-GEN.23103289 | PV428808 |
| SUB1522736816S-C-O-2-SHUI.23103271 | PV428809 |
| SUB1522736816S-C-O-1-GEN.23103275 | PV428810 |
| SUB1522736816S-CF1-23-1.23047910 | PV428811 |
| SUB1522736816S-CF1-23.23103285 | PV428812 |
| SUB1522736816S-CF1-22.23047900 | PV428813 |
| SUB1522736816S-CF1-19.23047914 | PV428814 |
| SUB1522736816S-CF1-18.22954786 | PV428815 |
| SUB1522736816S-CF1-17-1.22954784 | PV428816 |
| SUB1522736816S-CF1-15.22954788 | PV428817 |
| SUB1522736816S-CF1-13.22954798 | PV428818 |
| SUB1522736816S-CF1-12.22954794 | PV428819 |
| SUB1522736816S-CF1-11.21548423 | PV428820 |
| SUB1522736816S-CF1-10.22954796 | PV428821 |
| SUB1522736816S-CF1-10.21566139 | PV428822 |
| SUB1522736816S-CF1-9.22954792 | PV428823 |
| SUB1522736816S-CF1-9.21548425 | PV428824 |
| SUB1522736816S-CF1-8-3.23072468 | PV428825 |
| SUB1522736816S-CF1-8.22954800 | PV428826 |
| SUB1522736816S-CF1-8.21548429 | PV428827 |
| SUB1522736816S-CF1-7.21548449 | PV428828 |
| SUB1522736816S-CF1-6.21548447 | PV428829 |
| SUB1522736816S-CF1-5.23103283 | PV428830 |
| SUB1522736816S-CF1-5.21548465 | PV428831 |
| SUB1522736816S-CF1-4.23047908 | PV428832 |
| SUB1522736816S-C-F1-3.21355268 | PV428833 |
| SUB1522736816S-CF1-2.23700747 | PV428834 |
| SUB1522736816S-C-F1-2.21355270 | PV428835 |
| SUB1522736816S-CF1-1.23700749 | PV428836 |
| SUB1522736816S-CF1-1.22824088 | PV428837 |
| SUB1522736816S-CF1-1.22824076 | PV428838 |
| SUB1522736816S-32.23720177 | PV428839 |
| SUB1522736816S-31.23720175 | PV428840 |
| SUB1522736816S-30.23720173 | PV428841 |
| SUB1522736816S-29.23720171 | PV428842 |
| SUB1522736816S-28.23720169 | PV428843 |
| SUB1522736816S-26.23720179 | PV428844 |
| SUB1522736816S-23.23720209 | PV428845 |
| SUB1522736816S-22.23720207 | PV428846 |
| SUB1522736816S-21.23720205 | PV428847 |
| SUB1522736816S-20.23720203 | PV428848 |
| SUB1522736816S-19.23720189 | PV428849 |
| SUB1522736816S-18.23740438 | PV428850 |
| SUB1522736816S-16.23720197 | PV428851 |
| SUB1522736816S-15.23720195 | PV428852 |
| SUB1522736816S-14.23720193 | PV428853 |
| SUB1522736816S-13.23720191 | PV428854 |
| SUB1522736816S-11.23720165 | PV428855 |
| SUB1522736816S-10.23720187 | PV428856 |
| SUB1522736816S-9.23720185 | PV428857 |
| SUB1522736816S-7.23720181 | PV428858 |
| SUB1522736816S-6.23720163 | PV428859 |
| SUB1522736816S-5.23720161 | PV428860 |
| SUB1522736816S-4.23720159 | PV428861 |
| SUB1522736816S-2.23720157 | PV428862 |
| SUB1522736816S-1-14.23739344 | PV428863 |
| SUB1522736816S-1-13.23739342 | PV428864 |
| SUB1522736816S-1-12.23739340 | PV428865 |
| SUB1522736816S-1-10.23739336 | PV428866 |
| SUB1522736816S-1-8.23739332 | PV428867 |
| SUB1522736816S-1-6.23739328 | PV428868 |
| SUB1522736816S-1-5.23739326 | PV428869 |
| SUB1522736816S-1-4.23739324 | PV428870 |
| SUB15229343FSR1.ITS1.22808243.B06.seq | PV426714 |
| SUB15229343SSR-5.seq | PV426715 |
| SUB15229343FSF-1.seq | PV426716 |
| SUB15229343FSF-2.seq | PV426717 |
| SUB15229343FSF-3.seq | PV426718 |
| SUB15229343FSF-4.seq | PV426719 |
| SUB15229343FSF-5.seq | PV426720 |
| SUB15229343FSR-1.seq | PV426721 |
| SUB15229343FSR-2.seq | PV426722 |
| SUB15229343SSF-1.seq | PV426723 |
| SUB15229343SSR-1.seq | PV426724 |
| SUB15229343SSR-2.seq | PV426725 |
| SUB15229343SSR-3.seq | PV426726 |
| SUB15229343SSR-4.seq | PV426727 |

**Supplementary Table 2** Network properties of bacterial and fungal communities in root zone of *Artemisia ordosica* Krasch.

|  | **Group** | **Number of nodes** | **Number of connections** | **Average degree** | **Average path length** | **Average weighted degree** | **Modularity** |
| --- | --- | --- | --- | --- | --- | --- | --- |
| **Bacteria** | C | 1131 | 4001 | 7.08 | 3.044 | 6.982 | 0.843 |
|  | SS | 103 | 3497 | 6.26 | 3.044 | 6.174 | 0.843 |
|  | FS | 113 | 1739 | 31.036 | 2.28 | 27.651 | 0.432 |
| **Fungi** | C | 642 | 7561 | 23.588 | 3.534 | 20.001 | 0.405 |
|  | SS | 483 | 7737 | 31.324 | 2.831 | 27.005 | 0.289 |
|  | FS | 806 | 9298 | 23.098 | 3.135 | 19.786 | 0.464 |

- 1. **Supplementary Figures**

**
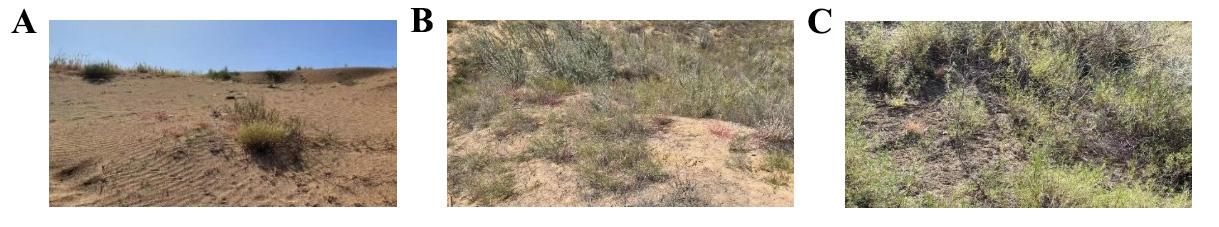
**

**Supplementary Figure 1.** Sampling sites of *Artemisia ordosica* Krasch. at different successional stages (A: Moving dunes (C); B: Semi-fixed dunes (SS); C: Fixed dunes (FS)).


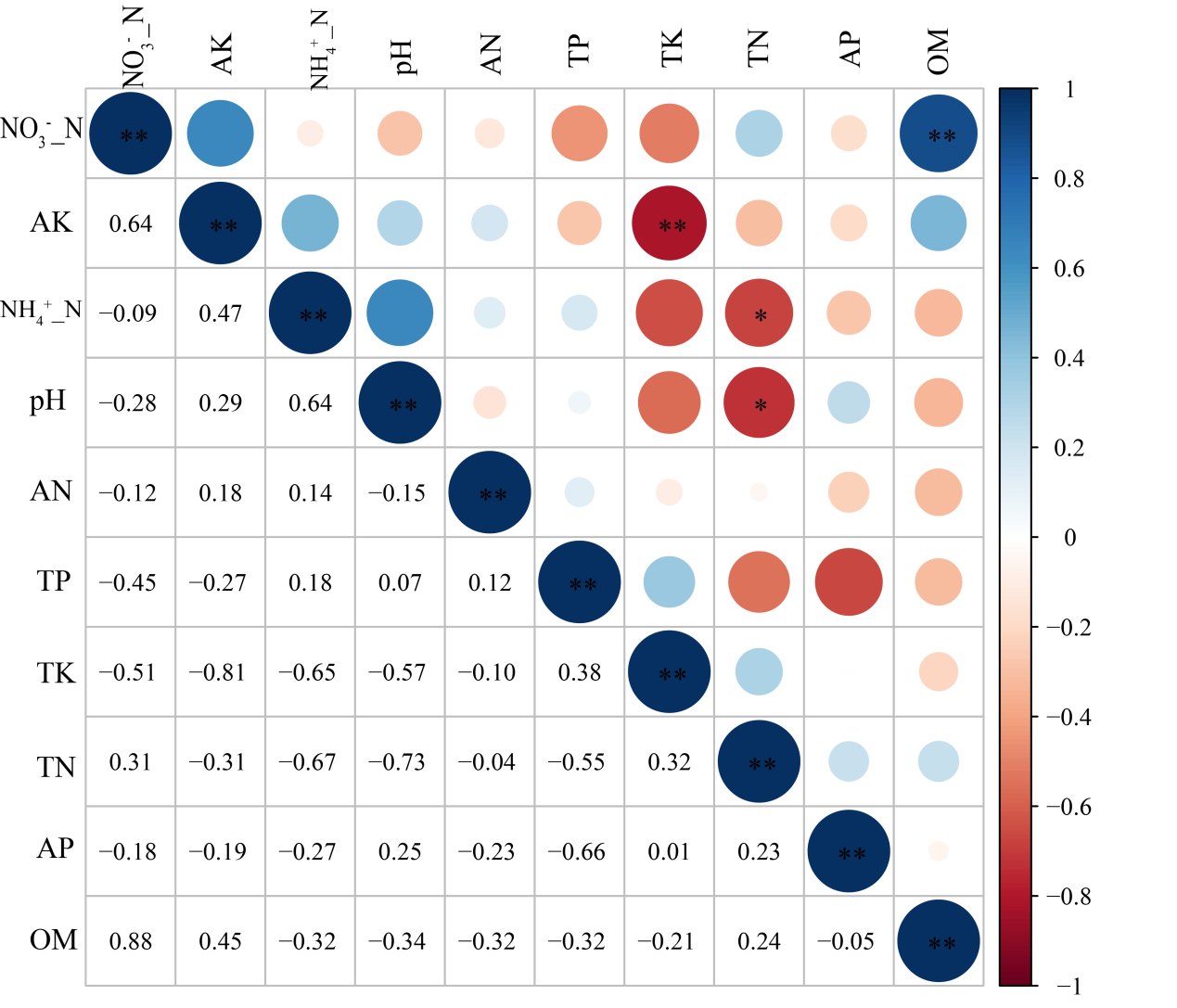


**Supplementary Figure 2.** Correlation Analysis of Soil Physicochemical Properties in the Root Zone of *Artemisia ordosica* Krasch. Rape. NH_4_^+^_N, ammonium nitrogen; OM, organic matter; AP, available phosphorus; TN, total nitrogen; TP, total phosphorus; TK, total potassium; NO_3_^-^_N, nitrate nitrogen; AK, available potassium; AN, available nitrogen; * indicates significance, * P <0.05, ** P ≤ 0.01.


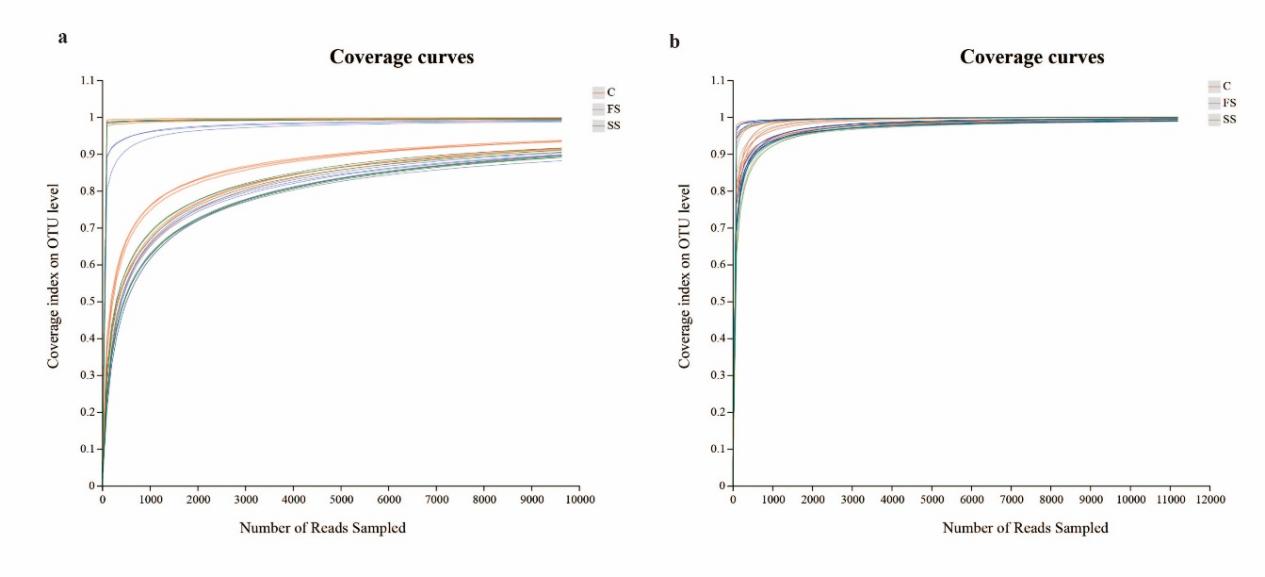


**Supplementary Figure 3.** The rarefaction curves analysis of the bacterial (A) and fungal (B) sequences. The curves were constructed using the Coverage index values of the OTUs and the number of reads.


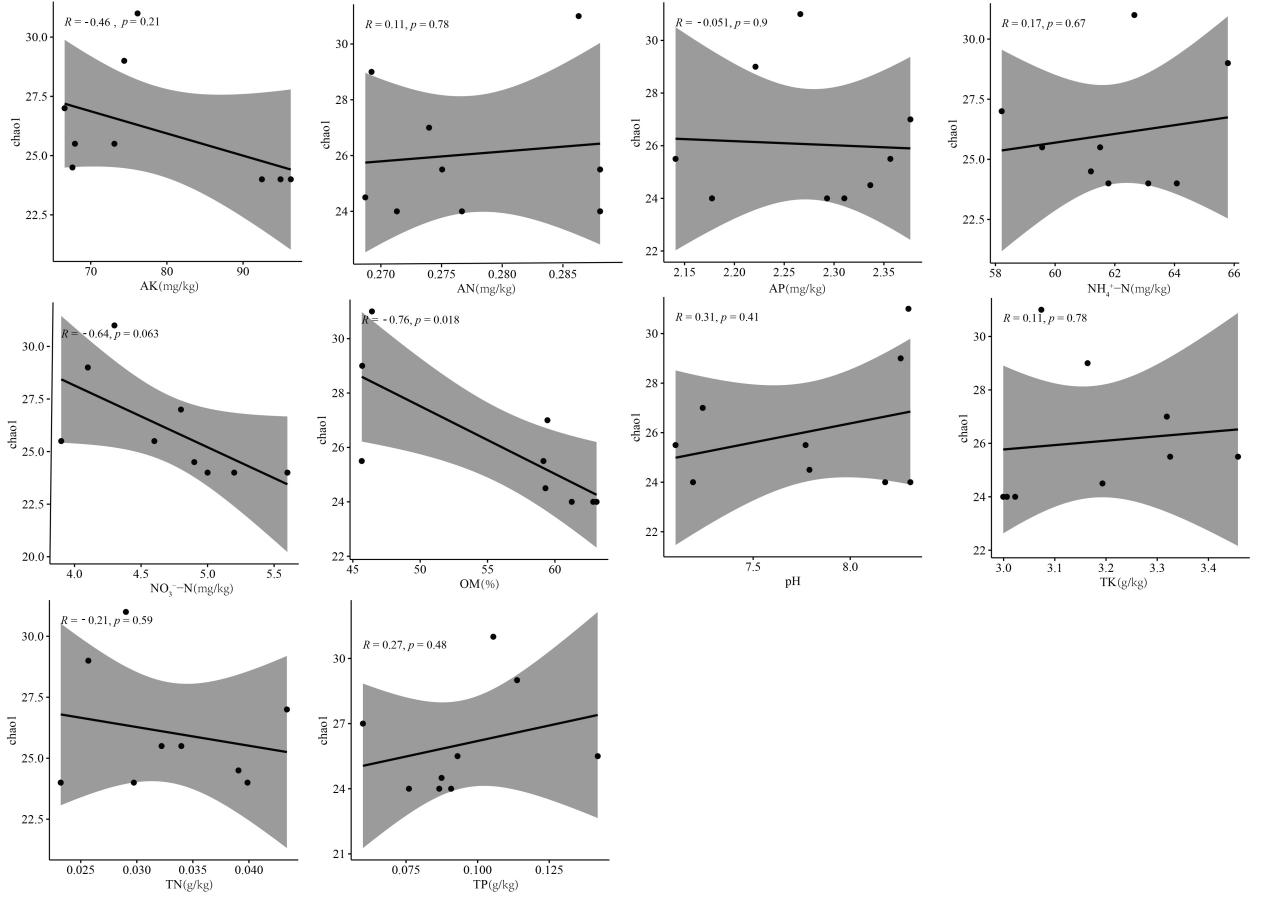


**Supplementary Figure 4.** Correlation analysis between soil physicochemical properties and Chao1 index of bacterial community. AK, available potassium; AN, available nitrogen; AP, available phosphorus; NH_4_^+^-N, ammonium nitrogen; NO_3_^-^-N, nitrate nitrogen; OM, organic matter; TK, total potassium; TN, total nitrogen; TP, total phosphorus.


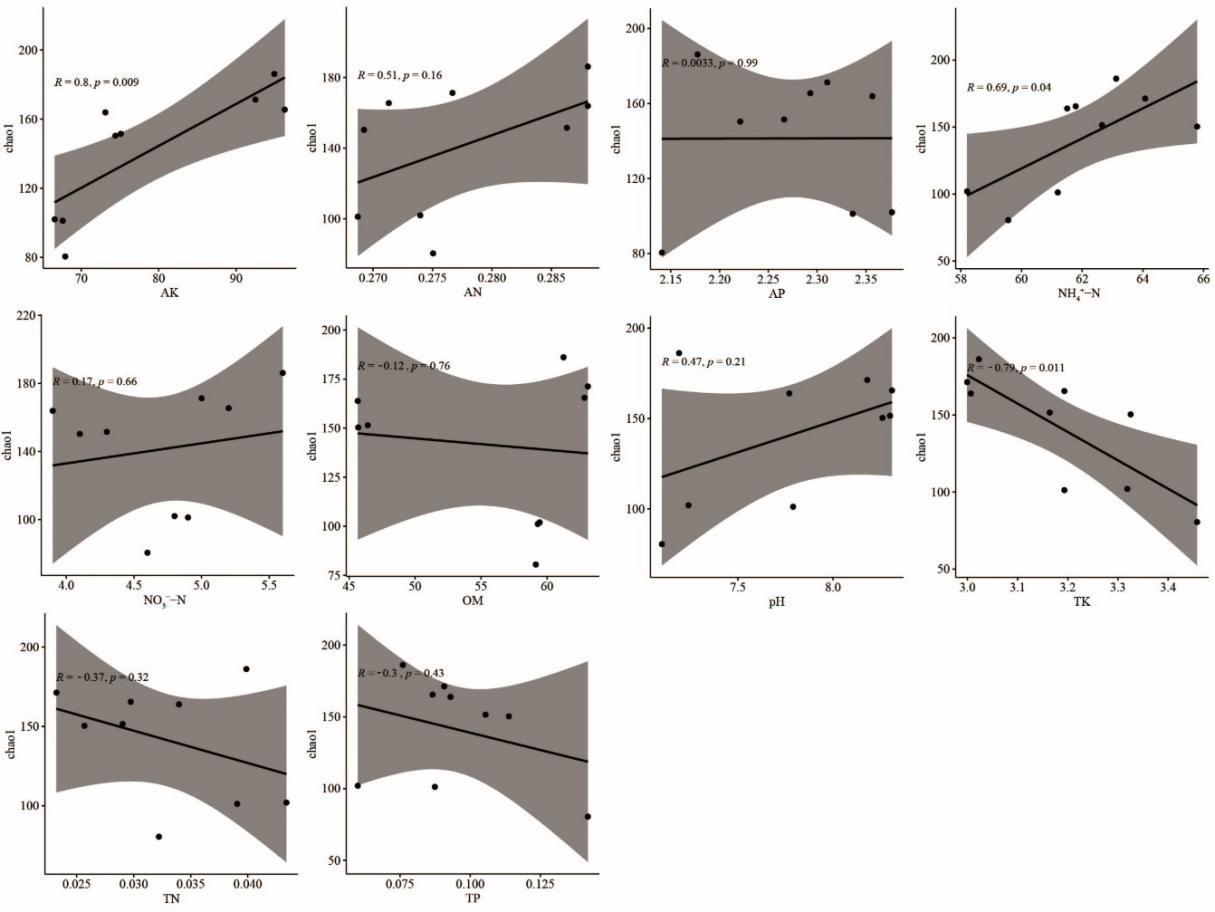


**Supplementary Figure 5.** Correlation analysis between soil physicochemical properties and Chao1 index of fungal community. AK, available potassium; AN, available nitrogen; AP, available phosphorus; NH_4_^+^-N, ammonium nitrogen; NO_3_^-^-N, nitrate nitrogen; OM, organic matter; TK, total potassium; TN, total nitrogen; TP, total phosphorus.


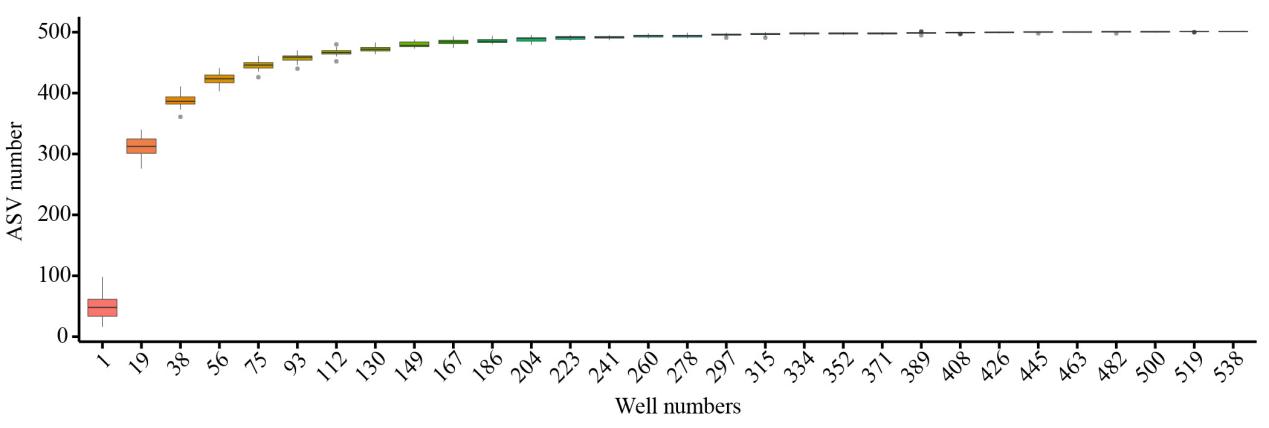


**Supplementary Figure 6.** The rarefaction curves analysis of microbial sequences in high-throughput culture in 96-well plates. The curves were constructed using the number of the ASV and the number of well.


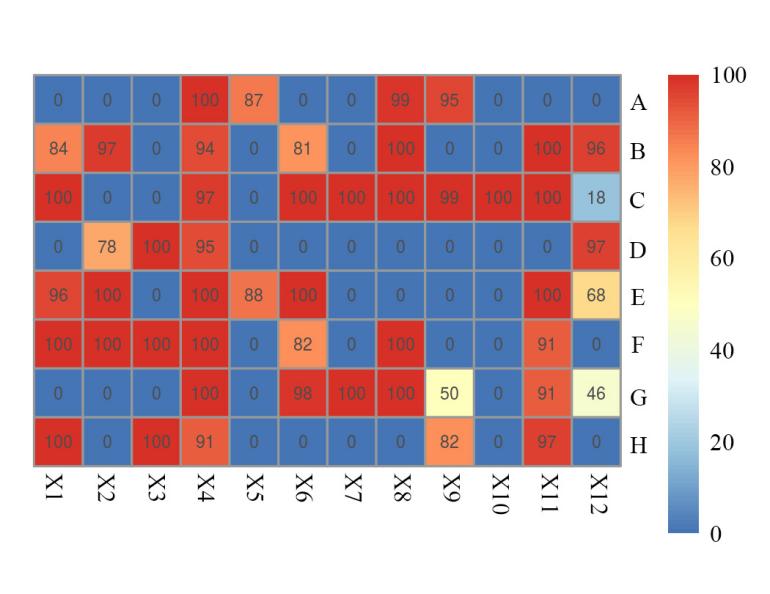


**Supplementary Figure 7.** Purity of the culture obtained in individual wells in a 96-well cell culture plate
